# Relative stability of chlorthalidone (CTD) Forms I and II: integrated experimental and theoretical insights into the superior thermodynamic stability of CTD-I driven by intra- and intermolecular interactions

**DOI:** 10.1007/s00894-026-07029-3

**Published:** 2026-10-30

**Authors:** Nathiya Singaravel, Jarede Da Silva Martins, Panneerselvam Murugesan, Juliana D. M. Silva, Leandro M. Santos, Antônio C. Doriguetto, Luciano T. Costa

**Affiliations:** 1 MolMod-CS - Instituto de Química, Universidade Federal Fluminense, Campus Valonguinho s/n, Centro, Niterói, Rio de Janeiro 24020-14 Brazil; 2 Laboratório de Cristalografia, Universidade Federal de Alfenas, Rua Gabriel Monteiro da Silva, 700, Alfenas, MG 37130-000 Brazil

**Keywords:** Chlorthalidone, Thermodynamic stability, Density functional theory (DFT), Solid-state analysis, Vibrational spectroscopy, Intra- and intermolecular interactions

## Abstract

**Context:**

Chlorthalidone (CTD), an antihypertensive drug, crystallizes in racemic polymorphic forms (Forms I and III) and as a conglomerate form (Form II). Understanding the relative stability of these crystal forms is essential for pharmaceutical solid-state characterization. Theoretical and experimental results consistently demonstrate the superior thermodynamic stability of Form I over Form II. Equilibrium solubility, thermogravimetric, and differential scanning calorimetry analyses support the computational findings. Calculated Raman and infrared spectra reproduce the main experimental features and reveal differences arising from distinct crystal packings. The results show that crystal stability is governed by a balance between intra- and intermolecular interactions, highlighting that the most stable crystal form does not necessarily correspond to the most stable isolated molecular conformer. These findings emphasize the importance of considering both molecular and packing effects in studies of pharmaceutical solids.

**Methods:**

All gas-phase calculations were performed in Gaussian09 using density functional theory (DFT) at the M06-2X/6–311 + G(d,p) level. Solid-state calculations were carried out with periodic DFT using the PBE-D3 functional in Quantum ESPRESSO. Crystal structures were modeled using plane-wave basis sets and projector augmented-wave pseudopotentials to investigate molecular conformations, crystal packing effects and relative stabilities. The bonding and intramolecular interactions were analyzed using QTAIM 2000 and Multiwfn software.

**Graphical Abstract:**

**Figure:**
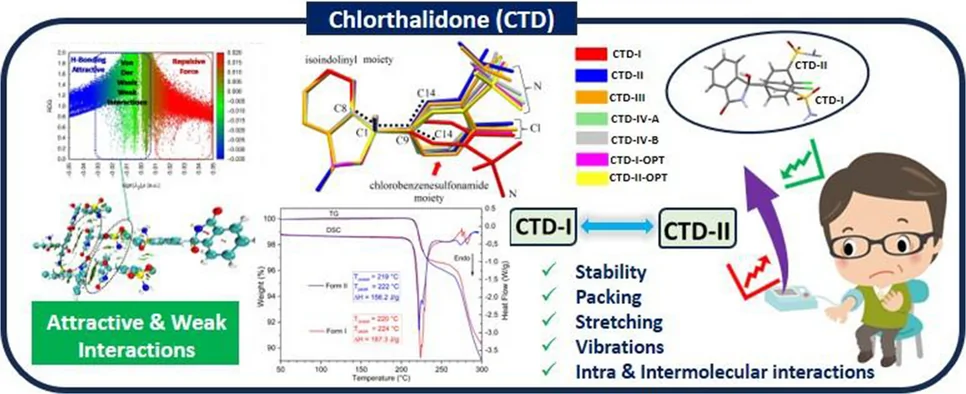

**Supplementary Information:**

The online version contains supplementary material available at https://doi.org/10.1007/s00894-026-07029-3.

## Introduction

Pure substances in the gas or liquid phase exhibit physicochemical properties mainly determined by their molecular features, such as atomic constitution, molecular geometry, and polarity. In the crystalline solid state, however, these properties also depend on the molecular arrangement within the crystal lattice and on the nature and strength of intermolecular interactions governing crystal packing [1]. Consequently, crystal packing and molecular conformation are intrinsically interdependent. Crystal structures emerge from a balance between intramolecular conformational preferences and intermolecular packing forces. Changes in molecular conformation modify the accessibility and spatial disposition of functional groups, thereby influencing intermolecular interactions and the resulting packing arrangement. Conversely, packing constraints may stabilize conformations that are not energetically preferred in isolation [1].

Active pharmaceutical ingredients (APIs) [2] frequently exhibit conformational flexibility arising from multiple rotatable σ-bonds. During crystallization, distinct conformations can generate different intermolecular interaction networks and packing arrangements, leading to polymorphism, which is defined as the ability of a solid substance to exist in more than one crystalline form while maintaining the same chemical composition [1, 3–6]. In flexible systems, polymorph selection is determined by the interplay between intramolecular conformational energetics and intermolecular lattice stabilization. Consequently, solid-state conformations do not necessarily correspond to minima on the isolated-molecule potential energy surface, as crystal packing may stabilize higher-energy conformers [4]. Solvent effects may further modulate conformational populations and nucleation pathways [6]. Therefore, polymorphism should be interpreted within the framework of the crystal energy landscape, where conformational, intermolecular, and lattice-energy contributions collectively define crystal-form stability.

Polymorphism is of particular relevance in pharmaceutical sciences because distinct crystalline forms may exhibit different physicochemical properties, including melting point, solubility, dissolution rate, and bioavailability. Despite extensive research, the occurrence of polymorphism remains difficult to predict solely from molecular structure, especially in flexible and chiral molecules [5–11].

The present study focuses on chlorthalidone (CTD), a chiral antihypertensive agent belonging to the thiazide-like diuretic class. CTD represents an attractive case study for solid-state investigations because reports of both polymorphic forms and enantiopure/racemic crystal forms indicate a complex crystal energy landscape, where conformational, packing, and chiral-recognition effects may contribute to the stabilization of competing crystalline phases [12–20]. The racemic crystal form, hereafter designated CTD Form I, was first structurally characterized by members of our group in 2009 [14]. This phase consists of an equimolar mixture of the two enantiomers and is currently recognized as the crystalline form conventionally specified for the API used in chlorthalidone pharmaceutical formulations [12–16]. Our interest in CTD was motivated by both the lack of crystallographic data for this API and literature reports describing two polymorphic forms, designated Forms I and II, based solely on powder X-ray diffraction (PXRD) and solid-state NMR analyses [17]. Our efforts to isolate these phases enabled the determination of the crystal structure of Form I and, unexpectedly, led to the discovery of a distinct crystalline phase, designated Form III [14]. Because Forms I and III are racemic phases with identical chemical compositions, Form III represents a true polymorph of Form I. Subsequently, in 2013, we determined the crystal structure of the phase recurrently detected in commercial raw materials and identified it as Form II [19]. Contrary to our initial hypothesis, Form II was not a true polymorph of Form I but rather the result of spontaneous enantiomeric resolution. This conclusion was supported by its crystallization in the chiral, non-centrosymmetric space group P2₁, which cannot accommodate a racemic crystal structure when Z′ = 1. Interestingly, crystallization produced a bulk material composed of a physical mixture of enantiomerically pure R and S crystals, constituting a conglomerate [19]. Collectively, these findings revealed the complexity of the CTD solid-state landscape and motivated a systematic investigation of the structural, energetic, and crystallization factors governing the formation, stability, and interconversion of its crystalline forms. In 2014, we reported the occurrence of multiple CTD crystalline forms in commercial raw materials and finished tablet formulations, including samples containing predominantly Form II or mixtures of Forms I and II [20]. That study established that Form II exhibits higher solubility and a markedly different dissolution profile compared with Form I, leading to the conclusion that variations in polymorphic composition may directly affect CTD bioavailability, with potential consequences for therapeutic efficacy and safety. Finally, in 2016, single-crystal X-ray diffraction conclusively confirmed the spontaneous resolution of CTD Form II, revealing both (R)- and (S)-enantiomers as inversion-twinned domains within the same crystal and establishing its conglomerate nature [21].

Regardless of their classification as polymorphs (Forms I and III) or racemic and enantiopure crystal forms (Form II versus Forms I and III), the known CTD crystalline forms exhibit distinct supramolecular arrangements and packing motifs that may affect their pharmaceutical properties. In particular, Form II displays significantly higher aqueous solubility than the commercial Form I [20–32], suggesting lower thermodynamic stability and weaker lattice stabilization. Consequently, Form I is expected to exhibit stronger intermolecular cohesion, higher melting point, and lower solubility. Nevertheless, quantitative relationships between crystal packing and the relative stability of these forms remain poorly understood.

Motivated by this question in this present work, a combined theoretical and experimental approach is employed to explore the relative stability of Form I and Form II. Form III was not included in the study, as all attempts to grow it either following the procedures from previous work [14] or employing other protocols were unsuccessful. Given that it has not been reproduced since its discovery and no additional occurrences have been reported, Form III may be considered a disappearing crystal form [33]. Therefore, the present study focuses exclusively on Forms I and II.

Theoretical calculations were performed for the isolated CTD molecule in the gas phase and for the packing structure based on prior Single-Crystal X-ray diffraction (SXRD) data, employing gas phase and solid-state Density functional theory (DFT) methods. Vibrational frequencies were analyzed and compared with calculated and experimental data obtained from Raman and IR spectral studies using DFT methods. Furthermore, experimental results from thermal analysis (presented in this work) and previously reported solubility assays [20] provide support for the theoretical findings. The study also examines the weak intermolecular interactions between the two forms and their influence on packing arrangements, as verified through non-covalent interaction (NCI) analysis [34] Hirshfield analysis [35] and quantum theory of atoms in molecules (QTAIM) [36] analyses.

## Experimental section

Form I was identified in CTD USP reference standard (100% purity, lot I0C255) purchased from United States Pharmacopoeia (Rockville, MD, USA). Form II was obtained from Form I (CTD USP reference standard) by hydrothermal methods as previously described [19]. The FTIR measurements were carried out using a Shimadzu Affinity^−1^ Fourier-transform infrared spectrophotometer. Spectra were recorded at room temperature over the 4000–400 cm⁻^1^ range in transmittance mode, with a spectral resolution of 4 cm⁻^1^ and 32 accumulated scans. Fourier-transform Raman (FT-Raman) spectroscopy was performed by means of a Bruker RFS27 instrument able to collect spectra with laser excitation at 1064 nm and coupled to a detector cooled with liquid N_2_. All spectra were carried out with 600 scans accumulated with a 2 cm^−1^ spectral resolution. Further, Powder X-ray diffraction (PXRD) data were collected using a Rigaku™ Ultima IV diffractometer equipped with Cu Kα radiation (λ = 1.5418 Å) and a graphite monochromator. Measurements were performed using a grooved glass sample holder at an operating voltage of 40 kV and a current of 30 mA. Data were acquired in step-scan mode over a 2θ range of 5–35°, with a step size of 0.02° and a counting time of 2 s per step. The experimental PXRD patterns were compared with simulated diffraction patterns generated from the CTD crystal structures deposited in the Cambridge Crystallographic Data Centre (CCDC) to confirm the phase identity of CTD Forms I and II (Figure S1). In addition, the thermogravimetric analysis (TGA) was performed using an SDT Q600 thermobalance (TA Instruments, New Castle, DE, USA) at a heating rate of 10 °C min⁻^1^ from room temperature (25 °C) to 300 °C under a nitrogen atmosphere (50 mL min⁻^1^). Differential scanning calorimetry (DSC) measurements were carried out using a DSC Q20 (TA Instruments, New Castle, DE, USA) under the same conditions. For both analyses, approximately 5.0 mg of each sample was placed in open alumina (TGA) or aluminum (DSC) crucibles.

## Computational methods

All gas phase computations were performed using the Gaussian09 software [37]. DFT calculations [38] were performed to optimize the geometries of all molecules in the gas phase using the hybrid M06-2X functional [39], with 54% HF, in conjunction with the standard triple-split valence 6–311 + G(d,p) basis set [40]. Frequency calculations showed no imaginary frequencies in the optimized structure, confirming that the geometries represent true minima. To gain insights into the vibrational characteristics of the molecule, IR and Raman analyses were carried out based on the optimized geometries. To evaluate the conformational flexibility of the isolated molecules, relaxed potential energy surface (PES) scans were performed for rotation about the C1–C9 bond using the M06-2X density functional. The torsional angle was varied from 0° to 360° in 10° increments, while all remaining geometric parameters were fully optimized at each constrained dihedral angle. The calculations were carried out using the 6-311G(d,p) basis set, which provides a reliable balance between computational efficiency and accuracy for organic molecular systems. To assess the basis-set dependence of the rotational energy profile, additional PES scans were performed at the M06-2X/aug-cc-pVDZ level. The relative energies obtained from the constrained optimizations were used to construct the rotational energy profiles and determine the rotational barriers (Figures S2–S3). Comparison of the two basis sets was performed to evaluate the robustness of the predicted torsional pathway and conformational preferences.

Furthermore, the QTAIM analysis was conducted using QTAIM 2000 software to investigate the bonding interactions [41]. In this context, all calculations were carried out at the same level of theory. Moreover, NCI analysis was conducted by using Multiwfn software [42], utilizing both packing structures and single-crystal geometries.

In addition, to study the effects of crystallization on the molecules, periodic density functional theory (DFT) calculations were performed using the Quantum ESPRESSO package [43]. Empirical dispersion correction (DFT-D3) was applied [44]. These calculations utilized the generalized gradient approximation (GGA) [45] with the Perdew–Burke–Ernzerhof (PBE) exchange–correlation functional. The single-particle wave functions were expanded using a plane-wave basis set, with a kinetic energy cutoff of 35 Ry and a charge-density cutoff of 280 Ry. The ion–electron interactions were modelled with the projector augmented wave (PWA) method. The lattice parameters obtained experimentally were considered, and all atomic positions were allowed to relax until the forces were reduced to below 25 meV/Å. To evaluate the lattice energy, a single-point calculation was performed for an isolated molecule extracted from the optimized crystal structure. The molecule was placed in a cubic simulation cell of 25 × 25 × 25 Å^3^ to avoid interactions between periodic images, and Brillouin zone sampling was restricted to the Γ-point. The lattice energy was calculated according to,

$${E}_{lattice }= \frac{{E}_{crystal}}{Z} - {E}_{molecule}$$

where (E_crystal_) is the total energy of the optimized crystal, (E_molecule_) is the total energy of the isolated molecule, and (Z) is the number of molecules in the crystallographic unit cell ((Z = 2)). Furthermore, intermolecular interaction energy analysis, Hirshfeld surface analysis, two-dimensional (2D) fingerprint plot generation and lattice energy calculations were conducted using CrystalExplorer at the CE-B3LYP/6-31G(d,p) level of theory [35]. A detailed description of the computational methodology employed for the lattice energy calculations is provided in the Supporting Information (SI).

## Results and discussion

**Scheme 1:**
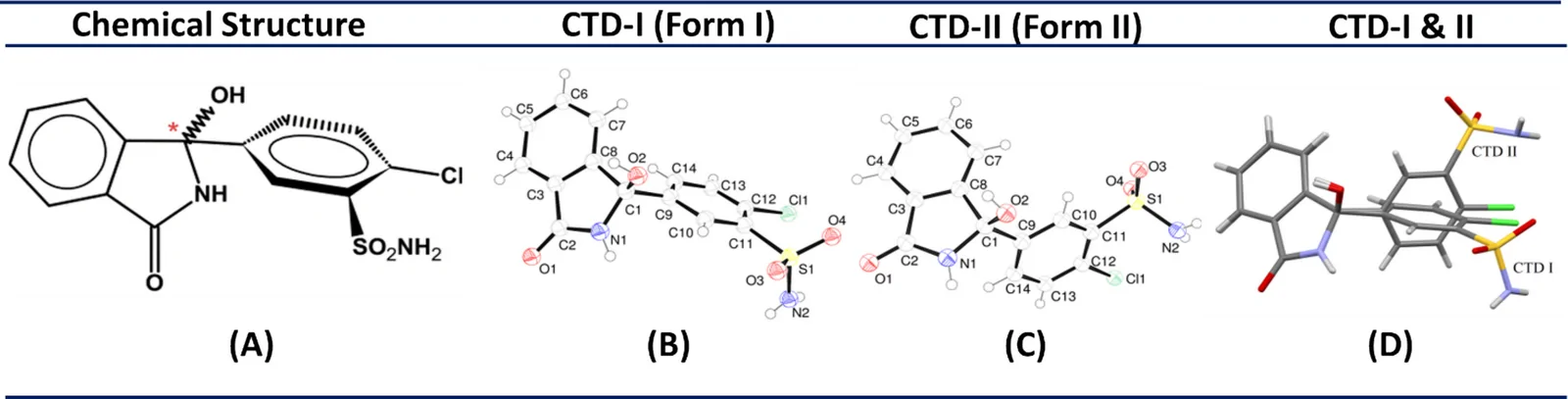
(**A**) Chemical structure representation of CTD**,** Ellipsoid plots with atom labeling of Form I (**B**) and Form II (**C**) conformers prepared from the CIF files YUCCIJ [14] and YUCCIJ03 [19] and the structure overlay (**D**) calculated through the isoindolinyl moiety. The original labeling scheme of Form I was modified making it equivalent to Form II. The R-enantiomer was arbitrarily chosen

Before discussing the results of the present study, a brief overview of the structural differences among the three known CTD crystal forms is warranted. In our previous studies [14, 18, 19], we showed that hydrogen bonding is the main stabilizing force in the crystal packing of CTD Forms I and II. Accordingly, we hypothesized [20, 46] that the greater stability of Form I relative to Form II arises primarily from these intermolecular interactions. Besides the distinct chiral composition of Forms, I (racemic) and II (enantiopure), CTD exhibits conformational flexibility about the C1–C9 bond (Scheme 1).

**Fig. 1:**
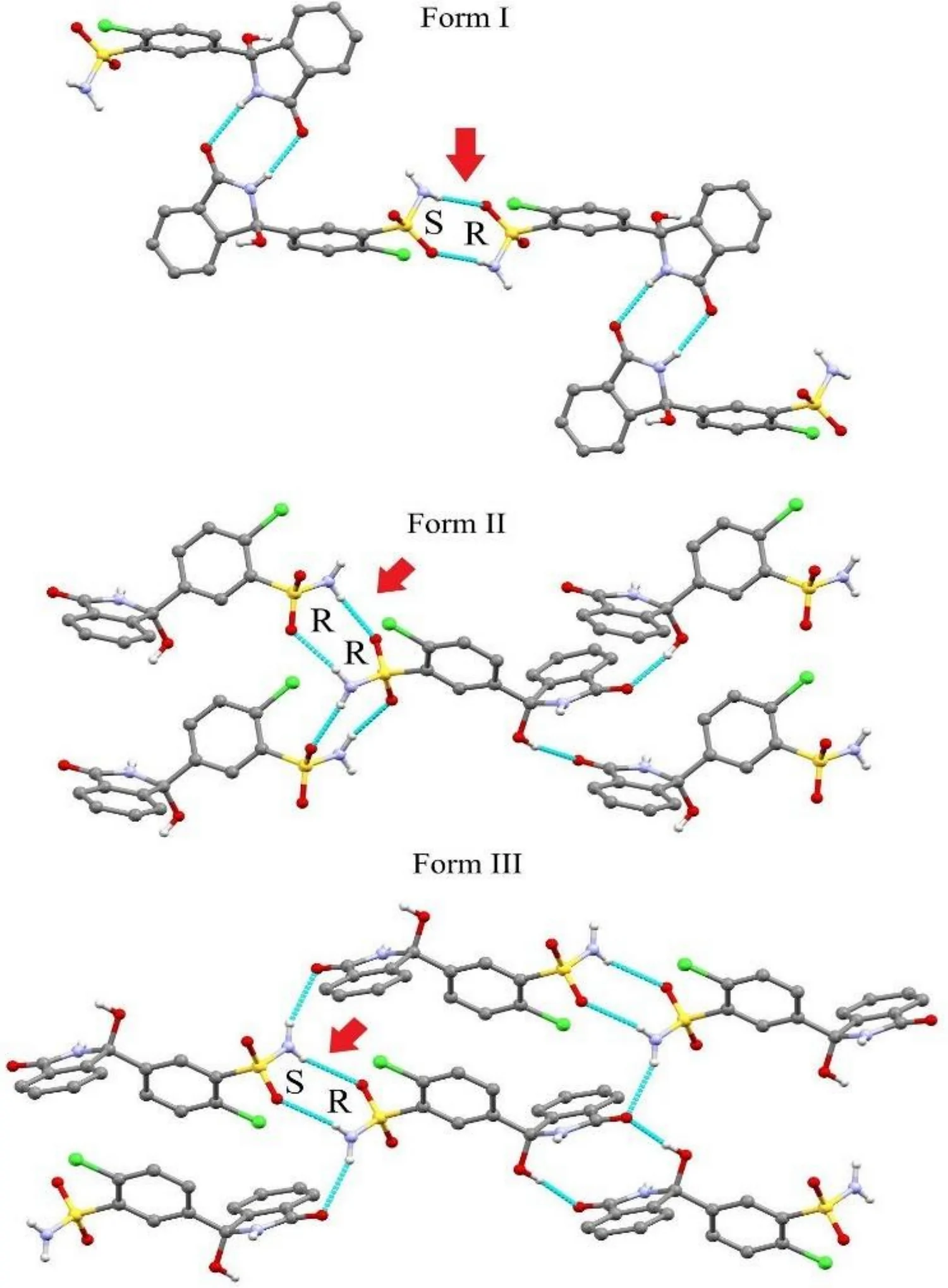
Partial view of the crystal packing of CTD Forms I, II, and III, highlighting the common supramolecular dimeric synthon formed by hydrogen bonds involving the sulfonamide groups (graph-set R22(8)), as well as the arrangement of enantiomers in the racemic (Forms I and III) and enantiopure (Form II) crystals. Hydrogen bonds are represented by cyan dashed lines. Hydrogen atoms bonded to carbon atoms have been omitted for clarity

Different torsional conformations lead to distinct hydrogen-bonding patterns and packing motifs, reflecting the interplay between molecular conformation, intermolecular interactions, and packing efficiency. The distinct hydrogen-bonding networks and enantiomer arrangements are highlighted in Fig. 1. Interestingly, Forms I, II, and III (the latter included for comparison because it is a racemic polymorph of Form I) all share the same sulfonamide R^2^_2_(8) synthon. In Forms I and III, this motif is generated by inversion symmetry, whereas in Form II it is generated by the 2_1_ screw axis, consistent with the P − 1 and P2₁ space groups, respectively. Another notable difference is the hydrogen-bonding pattern involving the isoindoline moiety, which plays a key role in defining the molecular conformation. Notably, Form III adopts a conformation similar to that of Form II, despite being racemic like Form I, suggesting that the hydrogen-bonding pattern has a greater influence on molecular conformation than the crystal's chiral composition. To provide a more quantitative and accurate explanation, first-principles electronic structure calculations based on DFT have been employed. DFT has proven to be a powerful tool for studying the structures of molecular crystals, providing valuable insights that complement experimental findings. In this study, we highlight the significance of accurate implementation and treatment of DFT methods to investigate the 3D structures of Form I and Form II. Furthermore, the relative stability of these crystal forms was evaluated to provide a clearer understanding and to support the previously reported experimental observations. In summary, this study aims to comprehensively examine the crystal forms, molecular conformation, packing stability, and thermal properties of the selected compounds using a combination of experimental techniques and theoretical calculations. Specifically, we analyzed the molecular geometry, vibrational spectroscopic features including IR, Raman spectra and structural characteristics. A particular focus was placed on understanding the nature and role of non-covalent interactions within these compounds through quantum chemical methods and periodic DFT calculations, further strengthening the connection between theory and experimental data.

### Solid state phase calculations

**Fig. 2:**
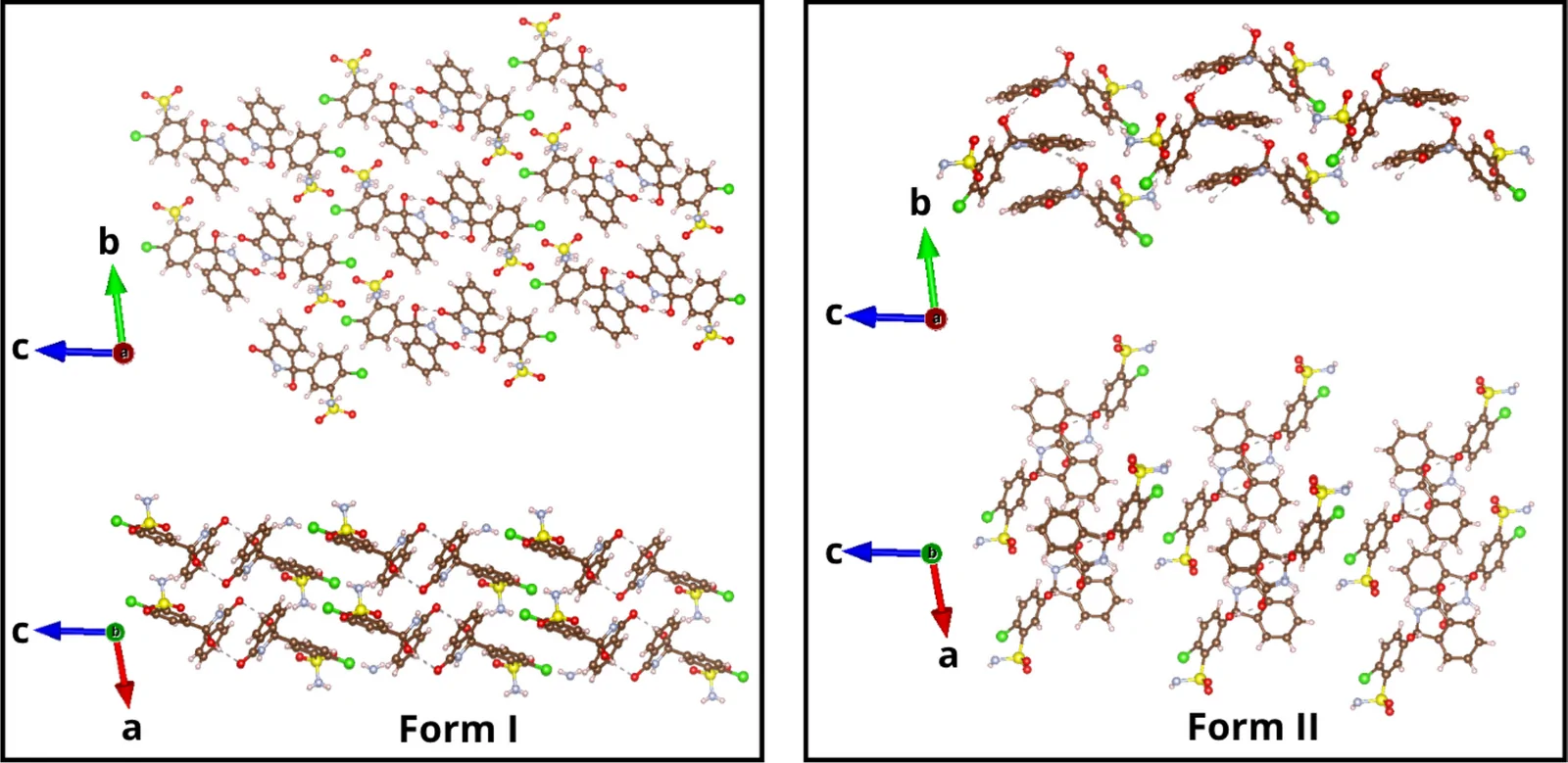
Representation of the different crystal packing arrangements of chlorthalidone forms viewed along the bc plane (top) and ac plane (bottom) for each structure. Carbon, oxygen, nitrogen, hydrogen, sulfur, and chlorine atoms are shown in brown, red, blue, white, yellow, and green, respectively

The crystal packing arrangements of chlorthalidone are shown in Fig. 2. CTD Forms I crystallize in the triclinic space group P-1, whereas Form II crystallizes in the monoclinic space group P2₁. For each structure, two packing views are presented: the bc plane in the top panel and the ac plane in the bottom panel. These visualizations clearly demonstrate that each crystalline form exhibits a distinct supramolecular arrangement and molecular packing organization. In addition, the crystal structures of CTD Forms I and II obtained from the CIF files were optimized using periodic DFT calculations, and the corresponding structural parameters are summarized in Table 1. The calculated results show good agreement with the experimental crystallographic data, indicating that the selected functional and dispersion correction constitute a suitable computational protocol for accurately describing the crystal structures. These findings are consistent with the results reported by Dubok and Rychkov [47], who demonstrated that the specification of appropriate parameters is essential for obtaining reliable thermodynamic and structural properties of organic crystals.

**Table 1:** Optimized parameters for Chlortalidone Forms I and II

	Form I			Form II		
Lattice Parameters	Exp	Cal	Rel. Error(%)	Exp	Cal	Rel. Error(%)
**a(Å)**	6.23	6.21	**0.32**	8.1	8.15	**0.62**
**b(Å)**	8.39	8.42	**0.36**	7.12	7.11	**0.14**
**c(Å)**	14.36	14.32	**0.28**	12.27	12.39	**0.98**
α(∘)	92.14	96.53	**4.76**	90	90	**-**
β(∘)	101.05	101.11	**0.06**	103.64	103.92	**0.27**
γ(∘)	107.02	107.06	**0.04**	90	90	**-**
Unit cell volume (Å^3^)	700.5	698.22	**0.32**	688.06	696.47	**1.22**

To evaluate the energetic contributions associated with the crystalline phases, periodic DFT calculations were performed for the optimized structures of Form I and II. Table 2 represents the total energies for both forms are reported in Rydberg (Ry). As shown in Table 2, the Form I exhibit a more negative total energy than Form II. The calculated relative energy difference (ΔE) is 0.73 kJ mol⁻^1^, indicating that Form I is slightly more stable than Form II. The small energy difference between Forms I and II is consistent with the literature, where ΔE values between polymorphic forms are typically reported to be below 4.184 kJ mol^−1^ [5]. However, as previously mentioned, Form II corresponds to an enantiopure crystal phase, whereas Forms I is racemic crystal forms, this fact indicates that their relationship is not strictly polymorphic.

**Table 2:** Total, relative and lattice energies of the optimized crystal structures obtained from periodic DFT calculations

Structure	E(Ry)^a^	ΔE (kJ/mol)^b^	E_lattice_(kJ/mol)^c^
Form I	−1279.46791028	0.0	−227.96
Form II	−1279.46735401	0.73	−217.75

^a^Raw total SCF energies obtained directly from the periodic DFT calculations; Ry is retained as the native unit of the calculation

^b^Relative energies (ΔE) were calculated using Form I as the reference. Conversion energy 1 Ry = 1312,75 kJ mol^−1^

^c^Lattice energy calculated according to the procedure described in computational methods

In this context, the lattice energy was also calculated to evaluate the relative stability of the two crystal forms. For true polymorphs, the energy differences are typically very small, generally less than 2 kJ mol^−1^ [18, 48, 49]. Consistent with the relative energy calculations, the lattice energy results indicate that Form I is thermodynamically more stable than Form II. The calculated lattice energy values (E_lattice_) are summarized in Table 2, revealing an energy difference of approximately 10.2 kJ mol^−1^ between the two structures. It should be noted that both the relative energies and lattice energies were obtained from periodic plane-wave DFT calculations at 0 K. Consequently, the experimentally observed stability may also be influenced by vibrational contributions, entropy, and kinetic effects, which are not included in the present calculations [18, 48]. Therefore, to further assess the relative stability of the forms under experimental conditions, thermal analyses (DSC and TG) were performed, providing complementary information on their phase behavior and thermal stability.

**Fig. 3:**
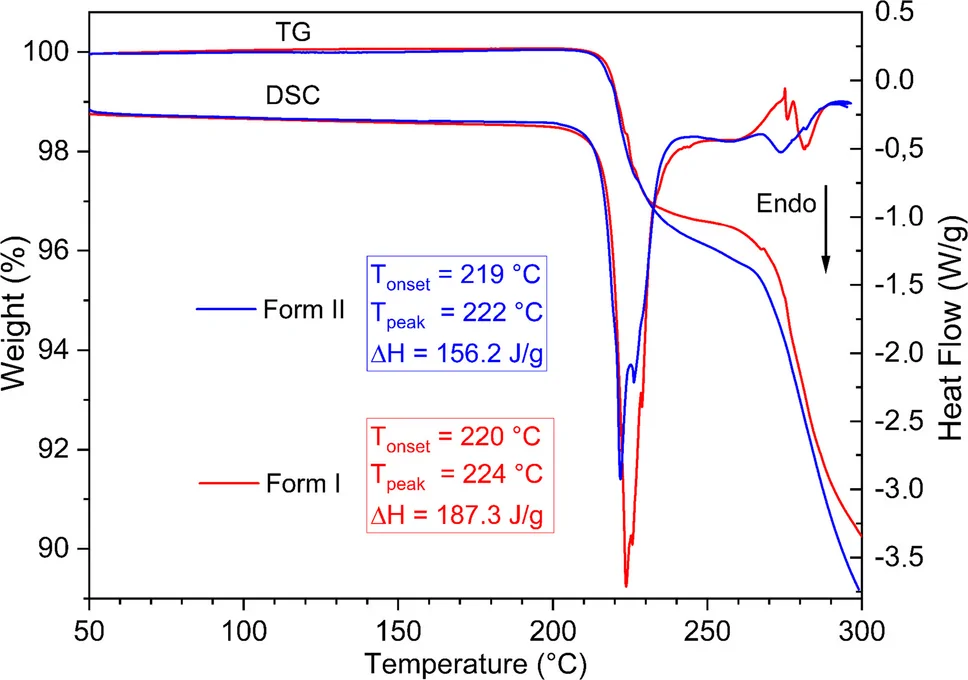
TG and DSC curves measured from 50 to 300 °C for Form I and Form II

The DSC curves of Forms I and II show thermal events at 224/220 °C (T_peak_/T_onset_) and 219/222 °C (T_peak_/T_onset_), respectively (Fig. 3). The corresponding TG curves reveal mass loss in the same temperature range, indicating that melting and decomposition occur simultaneously. As a result, the DSC endotherms cannot be attributed solely to melting, limiting a direct thermodynamic interpretation. Despite this limitation, Form II exhibits lower transition temperatures and a lower ΔH_fusion_ value (156.2 J g⁻^1^/53.0 kJ·mol⁻^1^) than Form I (187.3 J g⁻^1^/63.5 kJ·mol⁻^1^), consistent with its lower crystal stability. Together with the theoretical calculations presented here and previously reported solubility data [20], the thermal results support the higher thermodynamic stability of Form I. It should be emphasized, however, that the observed differences therefore reflect the behavior of distinct crystal phases rather than a polymorphic relationship [50]. Interestingly, Form II exhibits a smaller unit-cell volume (688.06 Å^3^) and a slightly higher crystal density (1.635 g cm⁻^3^) than the racemic Form I (691.727 Å^3^ and 1.626 g cm⁻^3^, respectively), when structures determined at the same temperature are compared (CCDC YUCCIJ02 for Form I and YUCCIJ03 for Form II). These values suggest a marginally more efficient molecular packing in Form II. However, the density difference is small and does not provide sufficient evidence to draw definitive conclusions regarding compliance with Wallach’s rule [51], which generally predicts higher densities for racemic crystals. Nevertheless, Form I exhibit a higher enthalpy of fusion, lower solubility, and greater thermodynamic stability, whereas Form II corresponds to a denser yet thermodynamically less stable crystal phase. These findings indicate that crystal stability in this system is governed not only by packing efficiency but also by the balance of intra- and intermolecular interactions [51–53].

### Molecular structural analysis

**Fig. 4:**
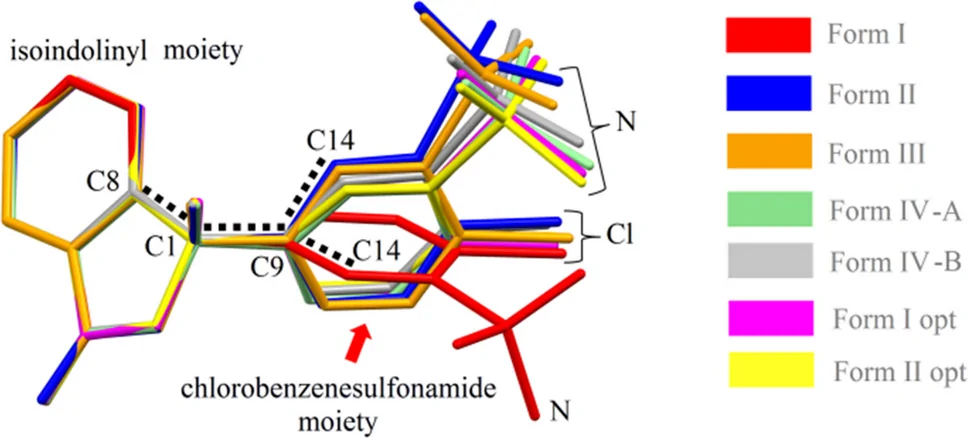
Overlay of optimized conformers (I & II) with experimental (I-IV) Form geometries highlights the homologous atoms C8-C1-C9-C14 (dotted lines) to distinguish the different orientations of the isoindolinyl and chlorobenzenesulfonamide moieties. Hydrogen atoms are omitted, with chlorine and nitrogen in the chlorobenzenesulfonamide moiety labeled for clarity

For the analyzed structures of Form I and Form II, which were optimized in their isolated forms, the structures differ in the orientation of the groups separated by the axis denoted by the atoms C8-C1-C9-C14 (Fig. 4). In the Form I, the chlorobenzenesulfonamide portion is oriented horizontally, while the isoindolinyl portion is oriented vertically. In contrast, for Form II, both portions are oriented vertically. Furthermore, Fig. 4 is an overlay view of the optimized various conformers in ab initio gas phase calculations with the experimental ones for the Form I (CCDC CIF file YUCCIJ [14]), Form II (CCDC CIF file YUCCIJ03 [19]), Form III; CCDC CIF file YUCCIJ01 [14]) and Form IV (CCDC CIF file GIBMIP [18]). It is highlighted that all calculated and experimental CTD conformers are matched, except for Form I experimental. Overall, Form I and Form II show differences in their individual molecular orientations and packing molecules, yet their conformer stabilities remain comparable in both individual and packing analyses when compared with all other conformers. Furthermore, Form I and Form II exhibit similar optimized geometric orientations at the M06-2X/6–311 + G(d,p) level of theory, as clearly illustrated in Fig. 4.

**Fig. 5:**
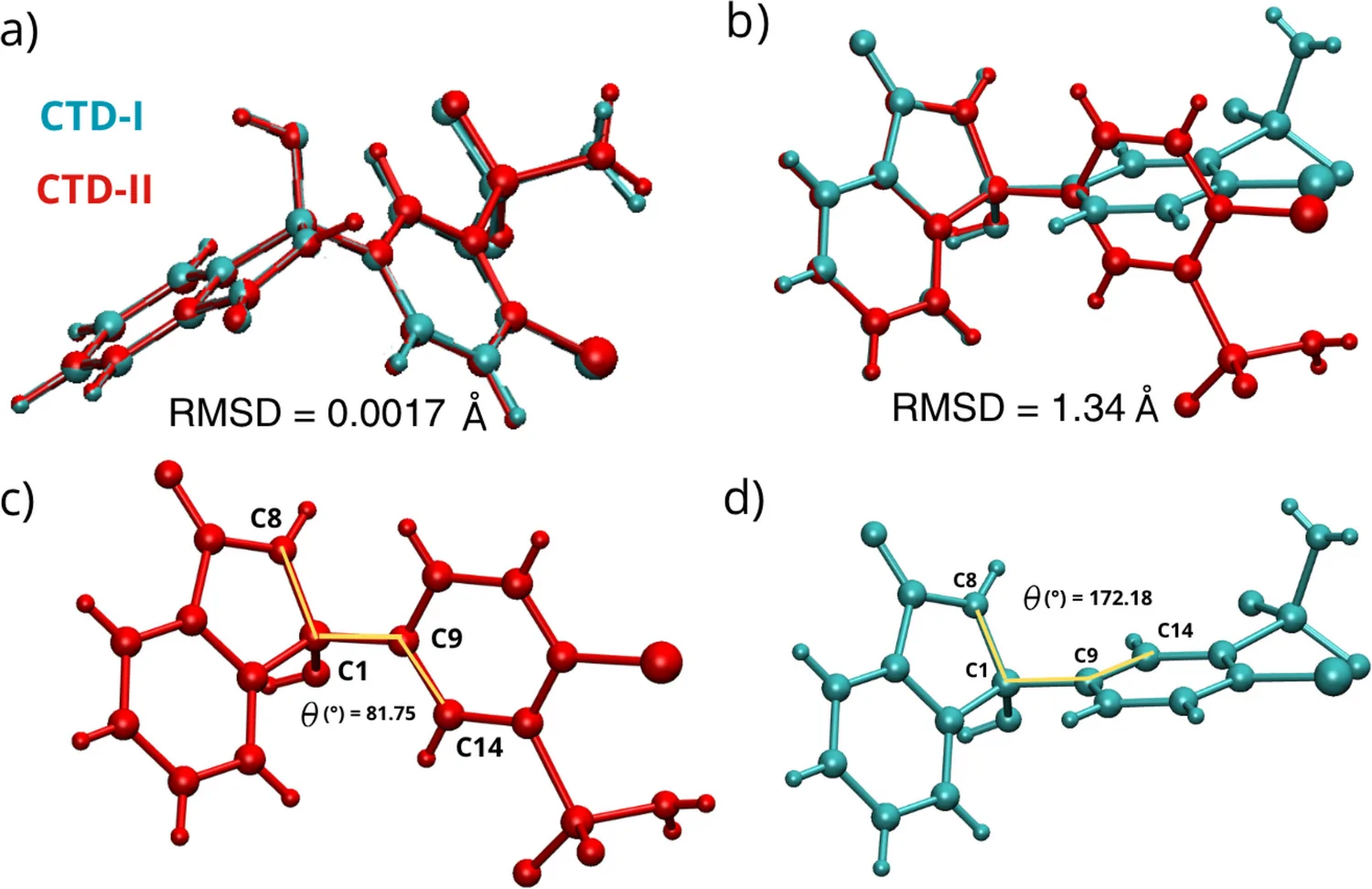
Structural comparison of CTD Forms I and II based on RMSD (**a**) isolated molecule optimized, RMSD*-gas;* (**b**) isolated molecule without optimization*, RMSD*-crys; (**c**) dihedral angle for Form II; and (**d**) dihedral angle for Form I

The significant difference in lattice energies between the two forms provides insight into the structural factors underlying their distinct stabilities. Accordingly, Cruz-Cabeza and Bernstein [4] investigated the structural relationship between the two forms using straightforward structural analyses commonly employed to compare different molecular conformations. These analyses included changes in torsion angles associated with rotational bonds and the root-mean-square deviation (RMSD) of atomic positions. It provides insight into whether the structural differences observed between the two crystal forms arise from distinct molecular conformations or from conformational adjustments induced by the crystal environment. In this regard, for the torsion-angle analysis, the molecules were isolated from their respective crystal structures, and the dihedral angle defined by atoms C8–C1–C9–C14 was determined. For Form I, the dihedral angle was 81.75°, whereas a value of 172.18° was obtained for Form II, as shown in Fig. 5. Thus, the maximum difference in the torsion angle, (Δθ_max_), was 90.43°. According to the classification proposed by Cruz-Cabeza and Bernstein, the angle within the range of 45° < (Δθ_max_) < 95° are not sufficient to distinguish between a conformational change and a conformational adjustment. Therefore, the analysis of individual rotational bonds is essential for understanding the nature of the structural differences between the two crystal forms. In this context, the RMSD analyses were performed for the two structural instances and are presented in Fig. 5. The molecular structures optimized in the gas phase are denoted as RMSD-gas, whereas the molecular conformations extracted directly from the corresponding crystal structures are denoted as RMSD-crys.

For the gas-phase structures, Form II exhibited a molecular orientation nearly identical to that of Form I, with an RMSD-gas value of approximately 0.0017 Å. The minor difference can be attributed primarily to the orientation of the amino-group hydrogen atoms, which adopted a opposite directions in the two optimized structures. This finding demonstrates that, upon removal of the crystal environment and subsequent gas-phase optimization, both molecular forms converge to essentially the same molecular geometry. In contrast, when the molecular conformations present in the crystal structures are considered, an RMSD-crys of 1.34 Å was obtained. The substantial difference between the RMSD values obtained for the gas-phase and crystalline structures indicates that the main structural discrepancy between Forms I and II is associated with the conformations adopted by the molecules within the crystal environment rather than with an intrinsic difference between their isolated molecular geometries.

Based on Cruz-Cabeza and Bernstein approach, the RMSD-gas below 0.1 Å are characteristic of pairs of conformers that differ mainly in the positions of hydrogen atoms. In this instance, the obtained value of 0.0017 Å falls well below this threshold. Therefore, in conjunction with the gas-phase optimization results, these findings indicate that Forms I and II do not represent intrinsically distinct molecular conformations in the isolated state. Conversely, the results are consistent with a conformational adjustment induced by the crystal environment, whereby the intermolecular interactions and crystal packing promote different molecular orientations in the two crystal forms. Accordingly, the observed differences between Forms I and II are more appropriately attributed to conformational adaptations arising from the crystal environment, rather than to intrinsically distinct molecular conformers. To further substantiate this explanation, the deformation energy was calculated using the lattice energies obtained from the DFT calculations. The lattice energy can be expressed as the sum of the deformation (E_def_) and intermolecular interaction (E_inter_) contributions as given by the following equation [54]:

$$E_{lattice}\;=\;E_{def}\;\;\;+\;E_{inter}$$

where E_def_ represents the energy required to distort the molecular geometry from its relaxed gas-phase structure to the geometry adopted in the crystal. Moreover, the E_def_ can be calculated as follows:

$$E_{def}\;\;=\;E_{crys}-\;E_{gas}$$

Thus, E_crys_ represents the energy of the molecule isolated from crystal without optimization, whereas E_gas_ accounts the energy for isolated molecule optimized. The values obtained from the above decomposition are summarized in Table 3.

**Table 3:** Calculated the lattice, deformation and intermolecular interaction energy. All values are given in kJ/mol

Structure	E_lattice_	E_def_	E_inter_
Form I	−227.96	18.69	−246.65
Form II	−217.75	11.89	−229.64

As outlined previously, the Form I exhibit a more negative lattice energy than Form II, indicating an overall stabilization of Form I. The decomposition of the lattice energy into deformation and intermolecular interaction contributions provides further insight into the origin of this difference. Form I present a higher deformation energy, with a value of 18.69 kJ mol^−1^, compared with 11.89 kJ mol^−1^ for Form II. This indicates that the molecular geometry adopted by Form I in the crystal incurs a larger energetic penalty relative to its relaxed gas-phase geometry. Interestingly, this higher deformation is compensated by more favorable intermolecular interactions in Form I. The calculated interaction energies are − 246.65 kJ mol^−1^ for Form I and − 229.64 kJ mol^−1^ for Form II. Therefore, despite the higher deformation energy associated with the molecular conformation of Form I, the intermolecular interactions established within its crystal packing contribute more substantially to its overall stabilization. The intermolecular interactions responsible for this conformational adjustment are discussed in the following sections, with particular emphasis on how the crystal packing and intermolecular contacts contribute to the stabilization of the different structures observed.

### Vibrational spectroscopy

**Fig. 6:**
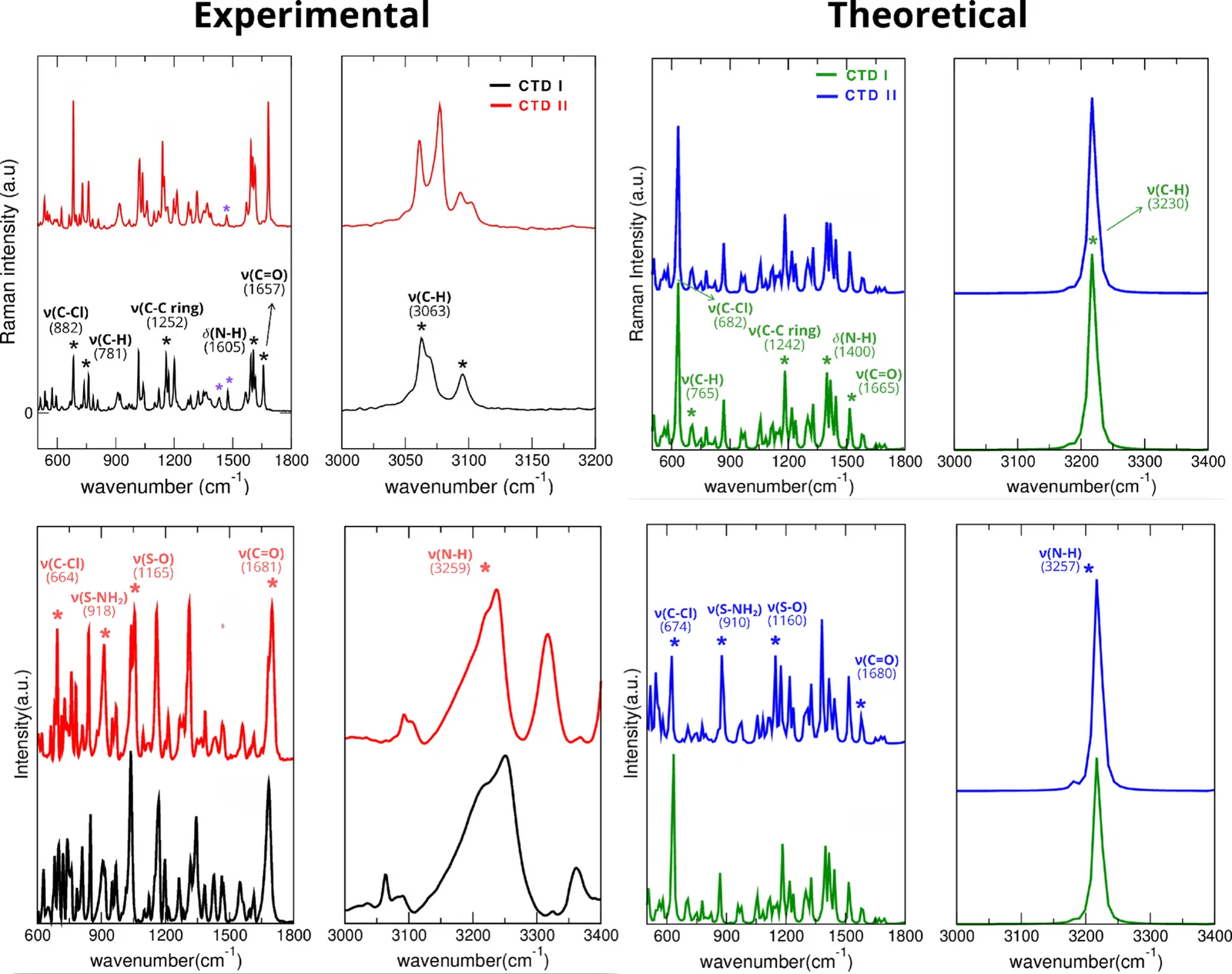
Comparison of the selected experimental FT-IR vibrational frequencies of the crystal packing system with the calculated IR spectra of the optimized single-molecule conformers (Form I and II) at the M06-2X/6–311 + G(d,p) level. The comparison between the experimental and theoretical IR spectra of the molecular packing system is presented in SI (Figure S5)

Experimentally, the investigation of the existence of the polymorphs can be performed using several techniques, such as microscopy, thermal analysis, spectroscopy, and X-ray diffraction, among others. This characterization is essential because polymorphs must be distinguished and classified according to all the criteria and parameters that define polymorphism. In addition to the thermal analyses (Fig. 3), we also experimentally obtained vibrational spectra for both forms and compared them with the theoretical results. Figure 6 compares the experimental and theoretical vibrational Raman and IR spectra of Form I and Form II. The experimental spectra (red and black) are displayed on the left, while the theoretical spectra (blue and green) are shown on the right. The upper graphs focus on the characteristic Raman vibrational modes, whereas the lower graphs highlight the infrared region. The theoretical Raman spectra of Form I and Form II show good agreement with the experimental spectra. Notably, the key peaks in the Raman spectrum of Form I align well with findings by Chagas and co-workers [55] (e.g., 1156 cm^−1^, 1605 cm^−1^, and 1657 cm^−1^). To further analyze the vibrational modes and the differences between the two crystalline forms, the experimental Raman spectrum highlights a region associated with symmetric and asymmetric CH bending of aromatic hydrogens (twisting), coupled with C–C stretching and N–H bending, around 1200 cm^−1^ to 1480 cm^−1^. Form I exhibit two experimental Raman bands at 1470 and 1484 cm⁻^1^, whereas Form II displays a single broad Raman band centered near 1458 cm⁻^1^. The corresponding calculated coupled vibrational modes of the crystal packing are illustrated in Figures S5 and S6, highlighting the vibrating molecular fragments and the intermolecular hydrogen-bonding network (yellow dashed lines) responsible for stabilizing the crystal packing. The calculated local vibrational mode of Form II appears at 1484.9 cm⁻^1^, while additional coupled modes are distributed over the 1406–1418 and 1467 cm⁻^1^ regions. The difference between the experimental and calculated frequencies is attributed to the systematic overestimation of harmonic DFT frequencies and vibrational coupling within the crystal environment. These results indicate that the distinct hydrogen-bonding environments modify the vibrational coupling, leading to band splitting in Form I and a single dominant Raman feature in Form II (Figures S5 and S6). The observed differences correlate with the distinct molecular packing of Form I compared to Form II, as shown in Figure S5 and S6. The contributions of individual vibrational modes and their associated peaks highlight the significant role of intramolecular hydrogen bonding in stabilizing Form I. However, these differences were not reflected in the theoretical Raman spectrum, which was derived using gas-phase DFT calculations of a pure system. The IR spectra demonstrated good agreement between the experimental and theoretical data. In the experimental spectrum, several key peaks were identified, including C–Cl stretching at 684 cm^−1^, S-NH_2_ stretching at 918 cm^−1^, and N–H stretching at 3259 cm^−1^. The theoretical spectrum highlighted two distinct regions, showing differences in vibrational modes between the two crystalline forms, particularly in bands with varying intensities, such as NH_2_ bending at 1470 cm^−1^ and C–Cl stretching at 684 cm^−1^. Consistent with the experimental Raman results, these variations reflect the unique molecular packing in Form I and Form II. Specifically, in Form I, the C–Cl stretching frequency exhibits higher intensity in both IR and Raman spectra due to intramolecular weak hydrogen bonding interactions (NH···Cl), indicating the significant stabilization provided by these interactions. Overall, the calculated IR and Raman spectral peaks align well with the experimental data for the packed systems. As shown in Fig. 6, the experimental peaks exhibit greater intensity, further emphasizing the critical role of intra- and intermolecular interactions in the packing of Form I and Form II systems.

### Intramolecular interaction characteristics: insights from NCI and QTAIM analysis

**Fig. 7:**
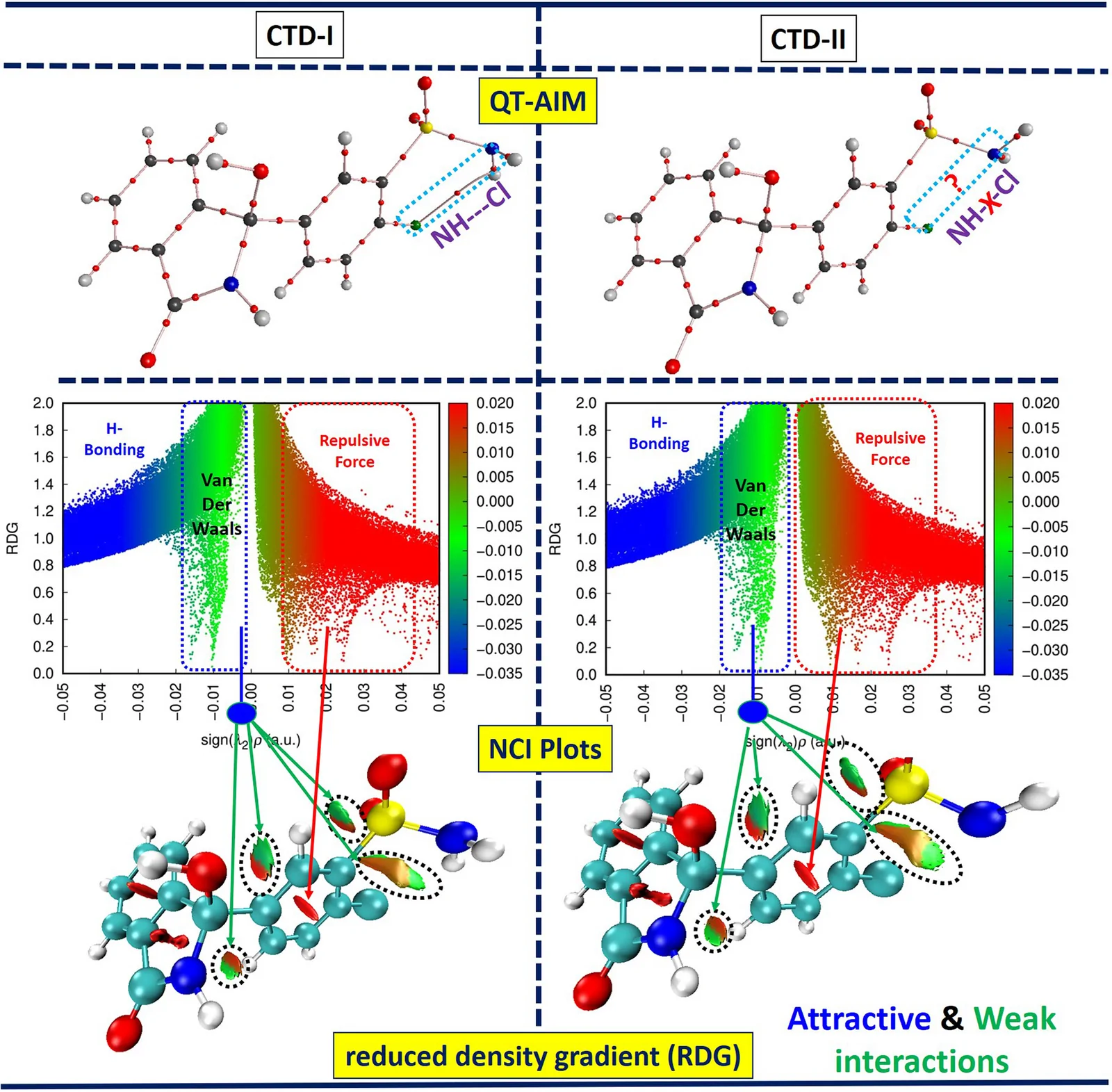
Topological properties (molecular graphs) from QTAIM analysis and RDG isosurfaces from NCI analysis were computed for the Form I and Form II conformers. The RDG isosurfaces were visualised at an isovalue of 0.5 a.u., with a color scale representing density values in the range of 0.1 < ρ < 0.1

**Fig. 8:**
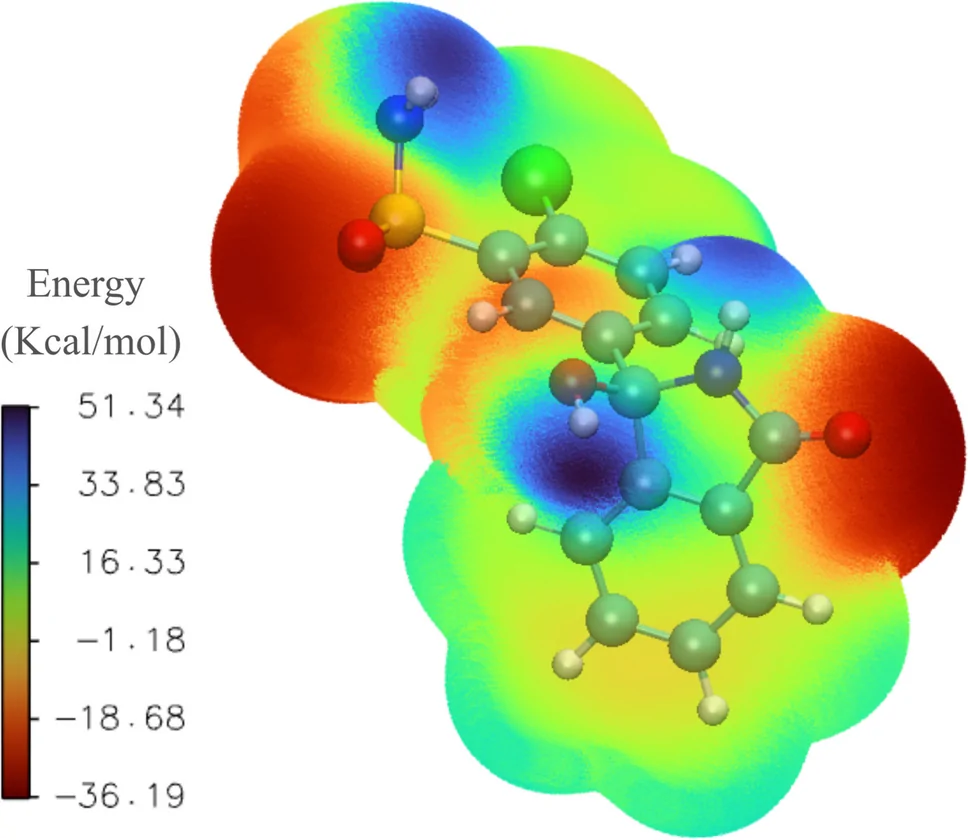
Electrostatic surface potential (ESP) mapping for Form I

Non-Covalent Interaction (NCI) analysis is an effective computational tool that helps to visualize and classify non-covalent interactions as attractive (blue), repulsive (red), and weak van der Waals (vdW) interactions (green) [34]. This study explores the nature and characteristics of intramolecular interactions using NCI analysis and the QTAIM. NCI analysis provides a visual representation of weak interactions, such as van der Waals forces and hydrogen bonds, by mapping reduced density gradients (RDG). QTAIM complements by identifying bond critical points and quantifying the electron density distribution, offering deeper insights into the strength and nature of these interactions. Together, these methods reveal how intramolecular forces contribute to the stabilization and geometry of molecular systems. Figure 7 illustrates the QTAIM topological properties and NCI analysis for the Form I and Form II geometries. The Form I exhibits a weak hydrogen bonding interaction (N–H···Cl), which is absent in Form II. To confirm the nature of this interaction, we illustrate how the charge density is distributed in these interactions with an electrostatic potential (ESP) analysis that was shown in Fig. 8.

Brammer et al*. *[56] states that the halogen atoms can act as hydrogen-bond acceptors (N–H···Cl), in which nitrogen is the hydrogen-bond donor and chlorine is the hydrogen-bond acceptor. In this context, the authors depict that in general the aforementioned type of interaction can be described like a weak hydrogen bond, is that D–H···X–C interactions have an attractive nature and are directional. In the region around the hydrogen, we observe a positive charge region and around the chlorine region a more negative charged region. Therefore, from the ESP analysis of Form I, we observed that although halogen atoms are involved, the N–H···Cl–C interactions observed were corresponds to weak hydrogen bond distance between (H···Cl) is 2.69 Å and angle (N–H···Cl ≅ 120.36°).

This interaction is identified as an intramolecular hydrogen bond that significantly influences the molecular packing in Form I. The NH···Cl interaction contributes to a more ordered packing arrangement in Form I compared to Form II, as evident from the molecular graphs obtained through QTAIM analysis. Furthermore, the NCI plot analysis provides clear evidence of the van der Waals weak interactions in Form I, as indicated by the sharp features in the RDG isosurfaces. In contrast, Form II lacks these specific interactions, showing only minimal and broad RDG isosurfaces, as depicted in Fig. 8. This distinct difference highlights the presence of possible weak hydrogen bonding intramolecular (NH···Cl) interaction in Form I, rather than Form II. Also, other weak interactions are found to be similar in both the packing structure conformers Form I and Form II.

### Packing analysis

**Fig. 9:**
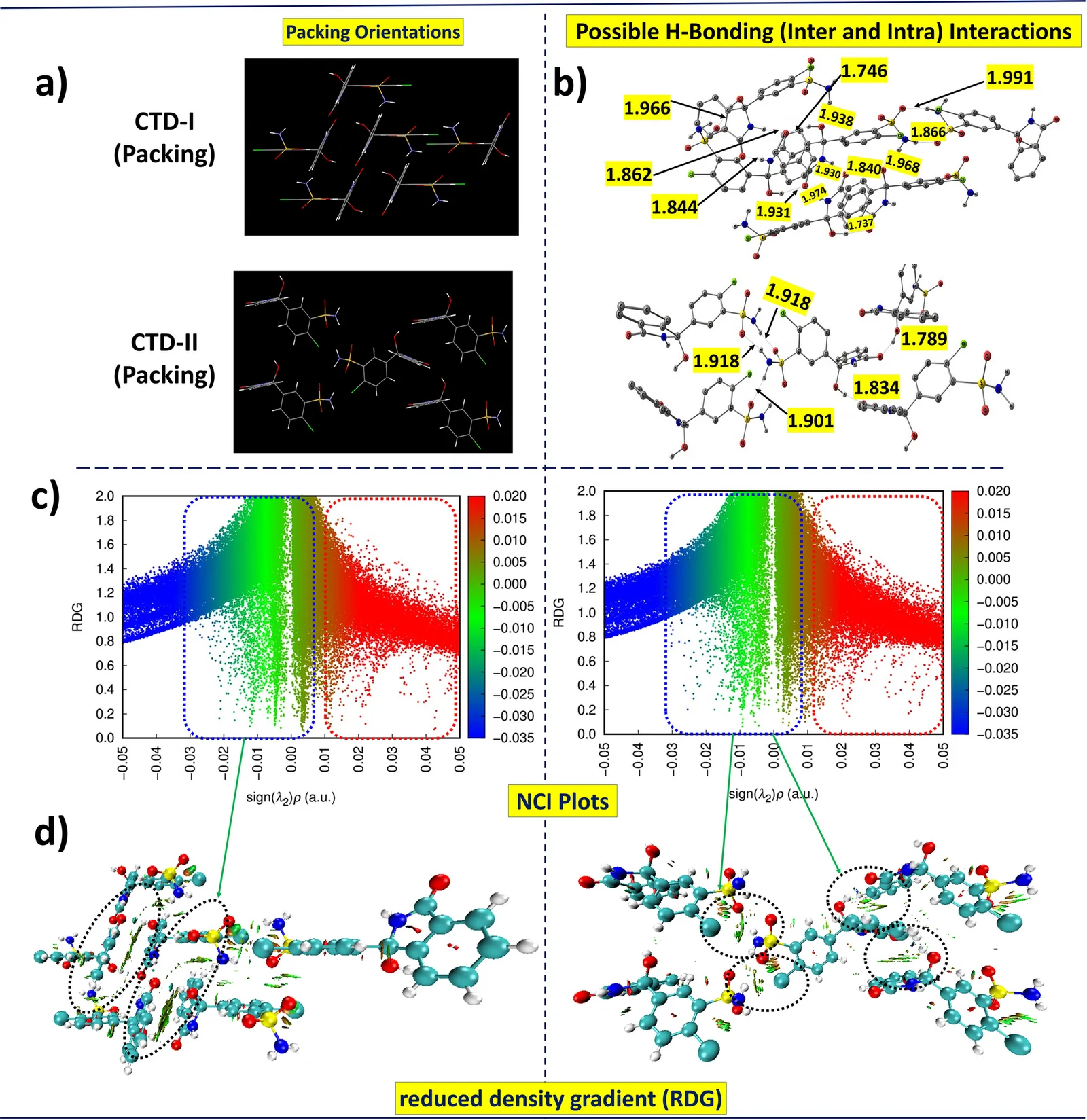
(**a**) Packing orientations for the Form I and Form II configurations and their possible H-bonding Interactions (**b**) were analyzed using experimental data, and RDG isosurfaces (**c** & **d**) from NCI analysis. The RDG isosurfaces were computed and visualized at an isovalue of 0.5 a.u., with a color scale representing density values in the range of 0.1 < *ρ* < 0

From Fig. 9a, the packing orientations of the Form I and Form II configurations, along with their potential H-bonding interactions (Fig. 9b), were analyzed using experimental data. Additionally, RDG isosurfaces (Fig. 9c and d) were generated from NCI analysis to further investigate the molecular interactions. The RDG isosurfaces were computed at an isovalue of 0.5 a.u. and visualized with a color scale, where the density values range from 0.1 to −0.1 (*ρ*), providing insight into the interaction strength and spatial distribution of the electron density around the molecular structures. The visualization indicates that Form I exhibits a more stable and optimal packing arrangement compared to Form II. This is attributed to the presence of both intermolecular and intramolecular hydrogen bonding interactions, along with a range of weak interactions, which collectively contribute to the more efficient packing in Form I. Figure 9b depicts the possible intermolecular and intramolecular hydrogen bonding interactions for both Form I and Form II. It further emphasizes the significantly higher number of these interactions present in Form I compared to Form II. In contrast, Form II shows only a limited number of interactions, approximately six, primarily involving N–H···O and O–H···O intermolecular hydrogen bonds. The stronger N–H···O interactions in Form I play a key role in stabilizing the packing structure, aided by both intermolecular and intramolecular hydrogen bonding interactions. To assess the impact of packing on the stability of each conformer, Fig. 9c and d present the results for the packing of Form I and Form II. Figure 9c displays the corresponding sign of (λ_2_)*ρ* values, while Fig. 9d shows an isosurface representing non-covalent interactions in the packing of both Form I and Form II conformers. The size of the blue and green isosurface in Fig. 9d clearly indicates significant interactions within the strong intramolecular packing interactions.

### Intermolecular interactions

**Fig. 10:**
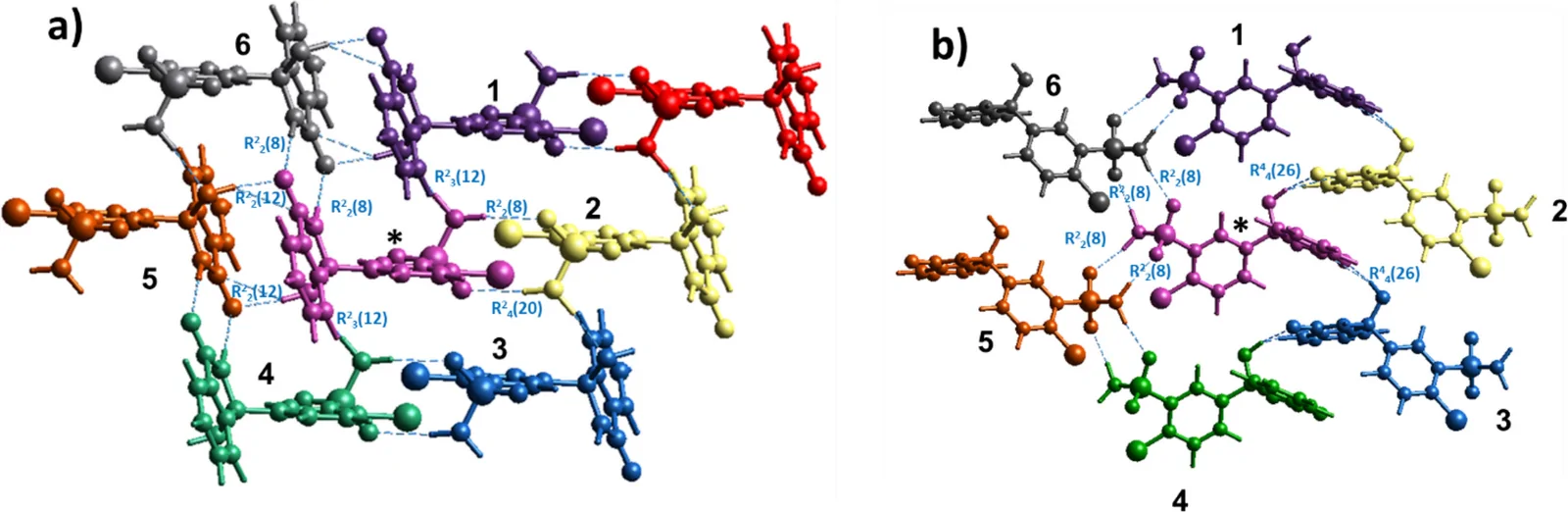
Representative hydrogen-bonding motifs and graph-set descriptors in CTD (**a**) Form I and (**b**) Form II

The total intermolecular interaction energies of Form I and Form II are presented in Figure S7. Six representative molecular pairs were selected for both Form I and II. Figure 10 presents the corresponding hydrogen-bonding motifs and their graph-set descriptors. The Form I, a total of eight classical hydrogen bonds are observed among the six representative molecular pairs, consistent with the previously reported crystal structure. The first molecular pair forms an R_3_^2^(12) ring motif through N–H···O and O–H···O hydrogen bonds. The second pair exhibit two hydrogen bonds involving N–H···O(SO_2_) and SO_2_···H–N interactions, generating an R_2_^2^(8) motif. The third pair does not form any classical hydrogen bond with the central CTD molecule. The fourth pair form a single O–H···N hydrogen bond, corresponding to an R_3_^3^(12) motif. The fifth pair forms two O–H···O = C hydrogen bonds, producing an R_2_^2^(12) ring. Finally, the sixth pair contain two N–H···O = C hydrogen bonds, giving rise to another R_2_^2^(8) motif. For Form II, a total of six classical hydrogen bonds is identified. Among the six representative molecular pairs, four pairs participate in classical hydrogen bonding. The second and third molecular pairs each exhibit O–H···O = C hydrogen bonds that generate an R_4_^4^(26) ring motif. The fifth and sixth pairs each form R_2_^2^(8) motifs through pairs of classical hydrogen bonds, contributing four hydrogen bonds in total. As a result, Form I exhibit additional hydrogen-bonding interactions, particularly pairs 1, 4, and 6, involving N–H···O and N–H···O = C contacts, whereas Form II is characterized only by SO_2_···N–H and O–H···O = C interactions. Therefore, these additional hydrogen-bonding interactions may contribute to the greater stabilization of Form I compared with Form II. The overall hydrogen-bonding network, comprising classical hydrogen bonds, is in good agreement with the hydrogen-bonding patterns reported in the previous literature. These hydrogen-bonding networks play an important role in stabilizing the crystal packing of both polymorphs.

### Hirshfeld surface analysis

**Fig. 11:**
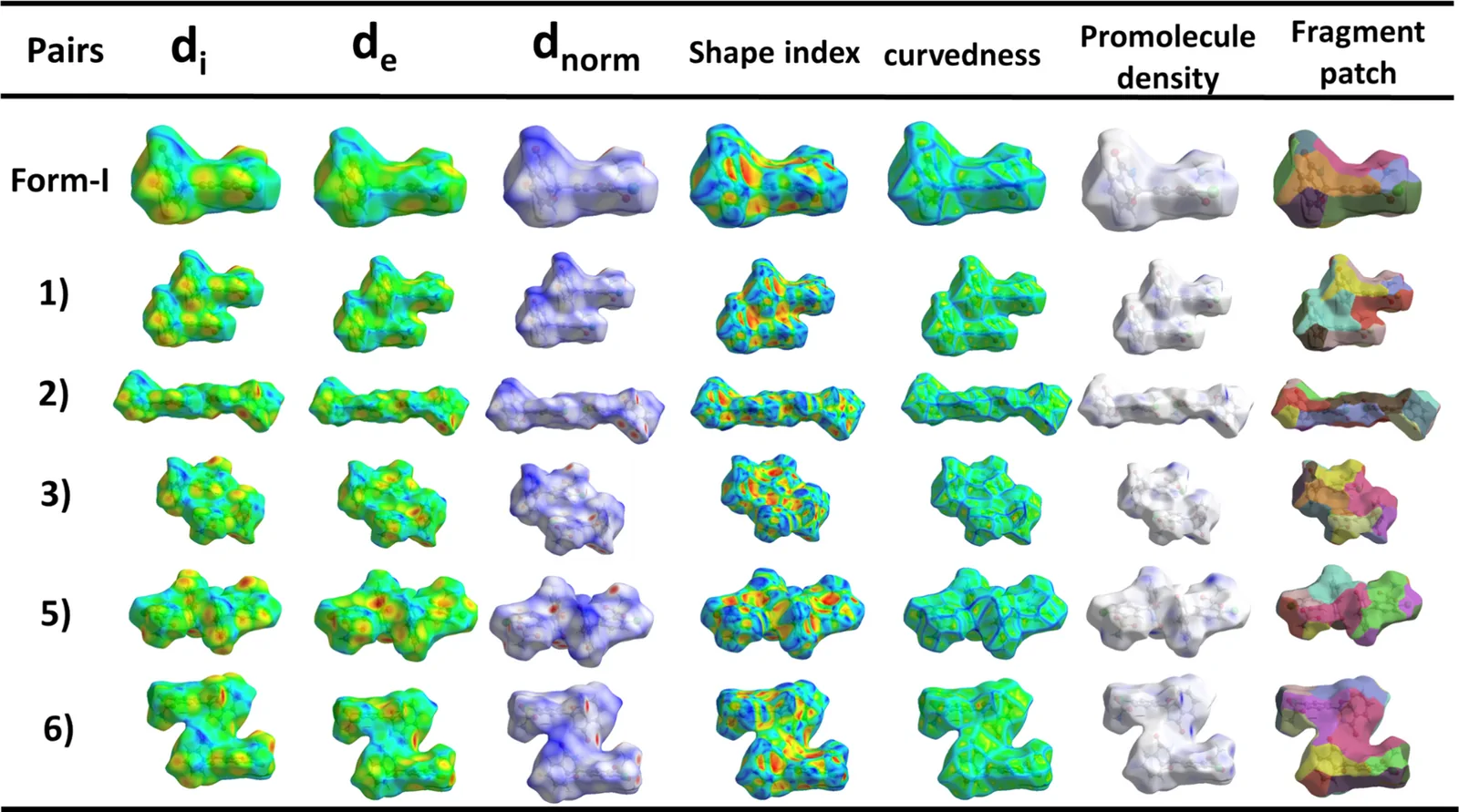
Hirshfeld surface representations of the representative molecular pairs of CTD Form I

**Fig. 12:**
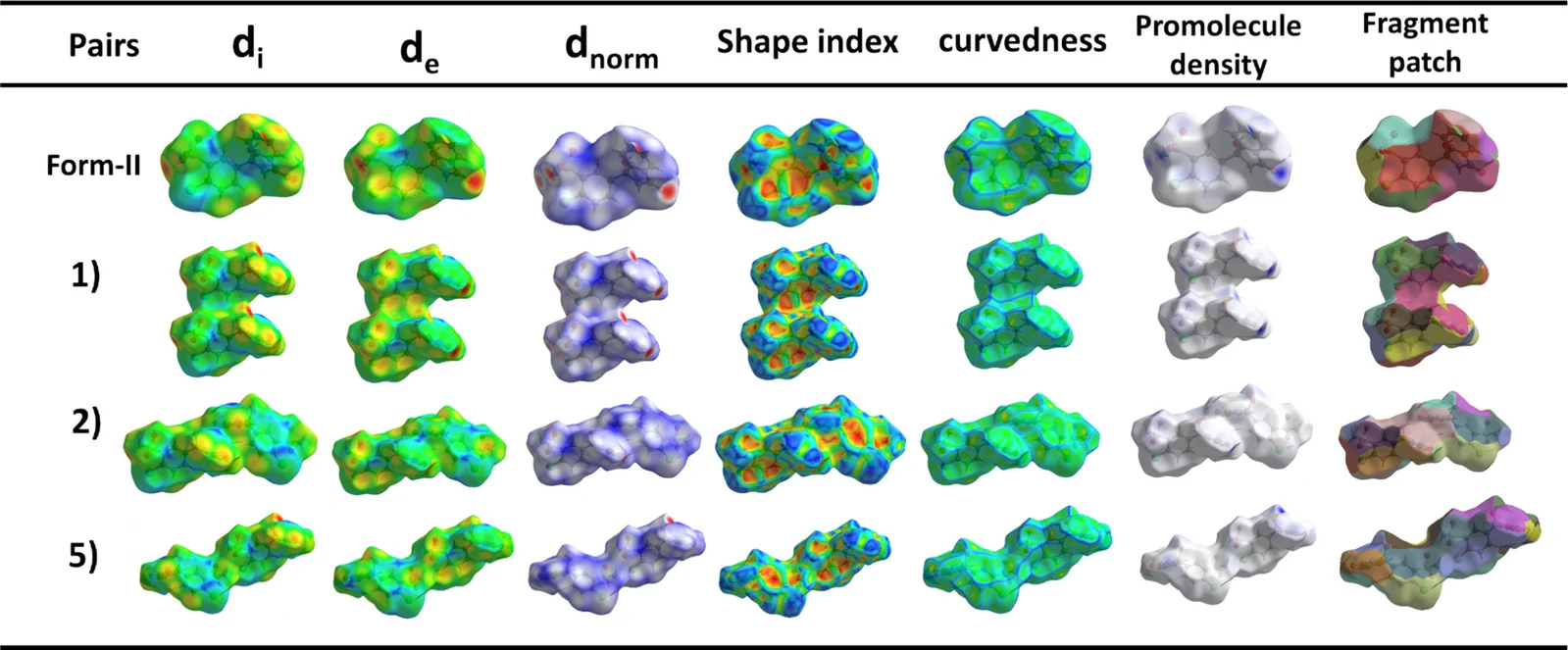
Hirshfeld surface representations of the representative molecular pairs of CTD Form II

The Hirshfeld surface analysis of both Form I and Form II was performed for molecular fragments and their corresponding dimers using the d_i_, d_e_, d_norm_, shape index, curvedness, promolecular density, fragment patch surface mappings, and the corresponding surfaces are presented in Figs. 11 and 12. In addition, five representative molecular pairs were selected for intermolecular interaction energy analysis in Form I, whereas three representative molecular pairs were analyzed for Form II. The d_i_, and d_e_ surfaces, which represent the distances from the Hirshfeld surface to the nearest atoms inside and outside the surface, respectively, collectively define the d_norm_ surface. The d_norm_ mapping reveals prominent bright red regions around the hydroxyl (O–H) and carbonyl (C = O) groups in both Form I and Form II, indicating short intermolecular contacts associated with strong hydrogen-bonding interactions that contribute significantly to crystal packing stabilization. The shape index surface is useful for identifying π···π stacking interactions. The presence of complementary alternating concave (red) and convex (blue) triangular regions on adjacent molecular surfaces suggests the existence of π···π stacking between neighboring aromatic rings. The curvedness surface complements the shape index by highlighting the local curvature of the Hirshfeld surface. Large, flat green regions indicate relatively planar surface areas that are favorable for π···π stacking, whereas blue regions correspond to highly curved portions of the surface. The promolecular density surface shows the distribution of the promolecular electron density, with blue regions observed around the hydroxyl group, reflecting its electron-density environment. The fragment patch surface divides the Hirshfeld surface into colored regions corresponding to different neighboring molecules, thereby illustrating the contributions of individual neighboring molecules to the intermolecular contacts surrounding the reference molecule. In addition, Hirshfeld surface analysis together with two-dimensional (2D) fingerprint plots was carried out to identify and quantify the different intermolecular contacts and their percentage contributions. The Hirshfeld surface mapped over the d_norm_ for CTD Forms I and II were performed for the corresponding dimers and molecular fragments are presented in Figures S8-S10. As can be seen from the Figure S8, the Cl···H intramolecular interaction makes a more predominant contribution in Form I than in Form II, which is consistent with the results obtained from the QTAIM and NCI analyses. For Form I, four representative molecular pairs were selected for intermolecular interaction analysis. The overall 2D fingerprint plot representing 100% of the Hirshfeld surface was initially analyzed by considering all intermolecular contacts. Furthermore, to evaluate the contribution of hydrogen-involved intermolecular interactions, the contacts involving hydrogen atoms with different atoms (H···X/X···H), including H···H interactions were analyzed accordingly. The Hirshfeld fingerprint plots for pairs 1 demonstrate that O···H/H···O contacts contribute the most significantly to the overall Hirshfeld surface (35.3%), followed by H··· H (24.7%), Cl···H/H···Cl (13.7%), N···H/H···N (1.2%) whereas S···H/H···S contacts show no contribution (0%). interactions. These results highlight the dominant role of O···H/H···O contacts in the intermolecular interaction network of Form I. A similar trend in the relative contribution of intermolecular contacts was observed for all the remaining molecular pairs, confirming the consistent nature of the interaction pattern throughout the crystal packing. For Form II, three representative pair interactions are presented in the Figure S10. Similar to Form I, O···H/H···O contacts show the highest contribution among the analyzed hydrogen-bond-related interactions, followed by other H-bond contacts. This consistent trend observed in both polymorphic forms indicates that O···H/H···O interactions are the most significant hydrogen-bonding interactions contributing to the intermolecular association in the crystal structures.

### Interactions energy analysis

The CE-B3LYP energy framework analysis decomposes the total interaction energy into four major components: electrostatic (E_ele_), polarization (E_pol_), dispersion (E_dis_), and exchange-repulsion (E_rep_) contributions. For the B3LYP/6-31G(d,p) electron density calculation, the scaling factors k_ele_, k_pol_, k_dis_, and k_rep_ were applied as 1.057, 0.740, 0.871, and 0.618, respectively. The calculated interaction energies for both polymorphic forms are summarized in the Table 4. In addition to the quantitative energy analysis, the intermolecular interactions were further categorized into structure-forming and non-structure-forming interactions to understand their contribution toward the stabilization and packing arrangement of the two crystal forms. Among the five representative molecular pairs analyzed for Form I, four were identified as SF interactions, of which three are hydrogen-bonded pairs. In contrast, Form II contains three representative SF interactions, including two hydrogen-bonded pairs. The strongest SF interaction in Form I exhibits a total interaction energy (E_tot_) of − 243.2 kJ mol^−1^ (E_ele_ =  − 208.8, E_pol_ =  − 52.8, E_dis_ =  − 134.3, and E_rep_ = 216.0 kJ mol^−1^), whereas the strongest SF interaction in Form II has a significantly lower stabilization energy of − 111.7 kJ mol^−1^(E_ele_ =  − 114.8, E_pol_ =  − 27.4, E_dis_ =  − 57.8, and E_rep_ = 129.9 kJ mol^−1^). The more negative interaction energy and larger electrostatic contribution in Form I indicate a stronger hydrogen-bonding compared with Form II. This observation is consistent with the Hirshfeld fingerprint analysis, in which O···H/H···O contacts exhibit the largest contribution among the hydrogen-bond-related interactions in both polymorphs, confirming that these hydrogen bonds are the principal structure-forming interactions governing the crystal packing. In addition, the lattice energy (E_lat_) of the crystal packing was calculated by summing the pairwise intermolecular interaction energies until the total lattice energy converged within 1 kJ mol^−1^(Figure S11). The calculated lattice energy values for Form I and Form II are summarized in Tables 4 and S1-S2. The lattice energies obtained from Quantum ESPRESSO are in good agreement with those calculated using CrystalExplorer, indicating consistent energetic trends between the two computational approaches. The yielded lattice energies of − 220.65 kJ mol⁻^1^ and − 212.10 kJ mol⁻^1^ for CTD Form I and Form II, respectively, whereas Quantum ESPRESSO predicted values of − 227.96 kJ mol⁻^1^ and − 217.75 kJ mol⁻^1^. Although the absolute values differ slightly, both methods consistently predict Form I to be more stable than Form II, with comparable energy differences between the two polymorphs.

**Table 4:** Intermolecular interaction energies of the selected molecular pairs and the calculated lattice energies (E_lat_) of CTD Forms I and II. All energy values are reported in kJ mol^−1^

Pairs	R	E_ele_	E_pol_	E_dis_	E_rep_	E_tot_	Bonding
Form I							
1	6.23	−16.1	−9.2	−49.9	41.8	−41.4	**HB**
2	10.95	−48.1	−8.9	−22.0	37.1	−53.7	**HB**
3	8.25	−7.7	−7.4	−23.9	18.3	−23.3	**-**
5	8.81	−76.5	−18.8	−45.2	86.2	−80.9	**HB**
6	8.03	−68.1	−15.9	−17.2	50.9	−67.2	**HB**
SF	34.02	−208.8	−52.8	−134.3	216	−243.2	**HB**
NSF	8.25	−7.7	−7.4	−23.9	18.3	−23.3	**-**
Lattice Energy (E_lat_)						**−220.65**	
Form II							
1	7.12	3.2	−1.0	−12.7	3.7	−6.1	**-**
2	8.95	−50.6	−15.0	−38.0	68.2	−55.5	**HB**
5	10.3	−64.2	−12.4	−19.8	61.7	−56.2	**HB**
SF	19.25	−114.8	−27.4	−57.8	129.9	−111.7	**HB**
NSF	7.12	3.2	−1.0	−12.7	3.7	−6.1	**-**
Lattice Energy (E_lat_)						**−212.10**	

Considering the overall findings from the analyses performed, the greater stability of Form I can be attributed to the nature and arrangement of its intermolecular interactions. Similar conformational adjustments associated with relatively high energy requirements for other molecular crystals, including PULNUF01 [57] and KUQLEO [58], for which conformational adjustment energies of approximately 11 kJ mol⁻^1^ have also been reported [4]. The analysis of the intermolecular interactions within the packing arrangement shown in Figs. 9b and 11 indicates that, in Form I, the molecule undergoes a conformational adjustment that facilitates the formation of two O–H···O = C hydrogen bonds between a pair of enantiomers, involving the hydroxyl and carbonyl groups. These interactions are characterized by short O···H distances of 1.862 Å and 1.844 Å and by highly directional geometries, with hydrogen-bond angles close to 177°. The amino group also contributes to the crystal stabilization through hydrogen-bonding interactions with the SO_2_ group (SO_2_···H–N) and the carbonyl group (N–H···O = C). However, these interactions are characterized by longer H···O distances of 1.866 Å and 1.968 Å, respectively.

In Form II, which contains only one enantiomer, a single type of O–H···O = C hydrogen bond is observed, characterized by an H···O distance of 1.789 Å and an O–H···O angle of 174°. Conversely, several interactions involving the amino groups and SO_2_ moieties are present, with the amino groups establishing bifurcated interactions with the sulfonyl oxygens. These interactions exhibit longer H···O distances, averaging ~ 1.918 Å, and hydrogen-bond angles of about 170°. In both forms, the SO_2_ groups adopt a trans conformation. However, their spatial arrangement differs considerably between the two crystal structures. In Form II, the SO_2_ and amino groups are arranged in alternating rows, allowing the two N–H hydrogen atoms to interact simultaneously with the oxygen atoms of neighboring SO_2_ groups. Conversely, in Form I, the amino and SO_2_ groups adopt a more aligned arrangement, while the trans-conformation of the SO_2_ groups is maintained. This difference in packing promotes the formation of the pair of the O–H···O = C hydrogen bonds between enantiomers in Form I. Thus, the conformational adjustment adopted by Form I, formed as an enantiomeric pair, produces a molecular geometry that favors a more favorable intermolecular hydrogen-bonding arrangement, compensating energetically for the structural deformation required to achieve this packing motif.

## Conclusion

The combined experimental and theoretical results indicate that Form I is more stable than Form II, primarily due to differences in their intermolecular packing. Although the isolated molecules show comparable electronic stability, Form I exhibit a more favorable network of hydrogen-bonding interactions and greater lattice stabilization. The deformation-energy analysis further indicates that the conformational adjustment adopted by Form I requires additional energy, which is compensated by the stronger and more favorable intermolecular interactions within its crystal packing. The QTAIM and NCI analyses further demonstrate the important role of both intermolecular and intramolecular hydrogen bonds in stabilizing its crystal structure. Overall, these results demonstrate that the relative stability of the chlorthalidone forms results from the balance between molecular conformation, deformation energy, and intermolecular interactions.

## Supplementary Information

Below is the link to the electronic supplementary material.

Supplementary file1 (DOCX 8859 kb)

## Data Availability

No datasets were generated or analysed during the current study.

## Declarations

### Data and computational files

The calculation files are available in the GitHub repository: [https://github.com/molmodcs/chlorthalidone-exp-theoretical-data].
