# Supplementary material for: Relative stability of chlorthalidone (CTD) Forms I and II: integrated experimental and theoretical insights into the superior thermodynamic stability of CTD-I driven by intra- and intermolecular interactions
Source: J Mol Model. 2026 Oct 30;32(11):454. doi: 10.1007/s00894-026-07029-3 (PMC13722741; doi:10.1007/s00894-026-07029-3)
Supplement: Supplementary file 1 [file 894_2026_7029_MOESM1_ESM.docx]

**Supporting Information**

**Relative Stability of Chlorthalidone (CTD) Polymorphs: Integrated Experimental and Theoretical Insights into the Superior Thermodynamic Stability of CTD-I Driven by Intra- and Intermolecular Interactions**

Singaravel Nathiya,^1#^ Jarede Da Silva Martins,^1#^ Murugesan Panneerselvam,^1^ Juliana D. M. Silva,^2^ Leandro M. Santos,^2^ Antônio C. Doriguetto^2^* and Luciano T. Costa^1*^

^1^MolMod-CS - Instituto de Química, Universidade Federal Fluminense, Campos Valonginho s/n, Centro, Niterói 24020-14, Rio de Janeiro, Brazil.

^2^Laboratório de Cristalografia, Universidade Federal de Alfenas, Rua Gabriel Monteiro da Silva, 700, 37130-000, Alfenas, MG, Brazil.

^#^*Contributed equally to this work*

**Corresponding Authors:* E-mail: [ltcosta@id.uff.br](mailto:ltcosta@id.uff.br) & doriguetto@unifal-mg.edu.br

**CONTENT CAPTIONS**

| **Figures** | | **Page No.** |
| --- | --- | --- |
| **Figure S1** | Experimental and calculated PXRD patterns of CTD Forms I and II. Calculated patterns were derived from CCDC entries YUCCIJ (Form I) and YUCCIJ03 (Form II). Asterisks mark reflections of CTD Form I in the experimental pattern of Form II, indicating a bulk phase mixture with Form I as the minor phase. | S2 |
| **Figure S2** | Potential energy profiles for the rotation about the C8-C1-C9-C14 bond in CTD-I (A) and CTD-II (B) computed at the M06-2X/6-311G(d,p) level of theory. The dihedral angle was varied from 0° to 360° in 10° intervals and nearly overlapping energy profiles (C) indicating similar conformational preferences and rotational flexibility for both compounds. | S3 |
| **Figure S3** | Rotational energy profiles of CTD-I and CTD-II obtained by scanning the C8-C1-C9-C14 dihedral angle (10° intervals) at the M06-2X/6-311G(d,p) and M06-2X/aug-cc-pVDZ levels of theory. Relative energies are reported in kcal mol-1, showing comparable rotational barriers and conformational trends for both basis sets. | S4 |
| **Figure S4** | Gas-phase optimized geometries of CTD-I and CTD-II, including their stable conformer energy differences (in kcal/mol) and possible intramolecular interactions for each conformer. | S4 |
| **Figure S5** | Experimental Raman spectra of CTD Form I (black line) and Form II (red line); theoretical Raman spectra of CTD Form I (green line) and Form II (blue line). | S5 |
| **Figure S6** | Visualization of the Raman-active vibrational modes in the ring-breathing region for CTD Form I (A) and CTD Form II (B). The highlighted molecular fragments represent the principal atomic displacements associated with the breathing vibrations, while the yellow dashed lines indicate the intermolecular hydrogen-bonding interactions that influence the vibrational coupling and crystal packing. | S6 |
| **Figure S7** | Representative intermolecular interaction molecular pairs of CTD (a) Form I and (b) Form II. | S7 |
| **Figure S8** | Two-dimensional (2D) fingerprint plots illustrating the overall and contributions of intramolecular hydrogen-bonding contacts in both forms. | S8 |
| **Figure S9** | Two-dimensional (2D) fingerprint plots illustrating the overall and contributions of intermolecular hydrogen-bonding contacts in CTD Forms I. | S9 |
| **Figure S10** | Two-dimensional (2D) fingerprint plots illustrating the overall and contributions of intermolecular hydrogen-bonding contacts in CTD Forms II. | S9 |
| **Figure S11** | Representative intermolecular molecular pairs within a 10 Å cutoff distance around the reference molecule for CTD (a) Form I and (b) Form II. | S10 |
| **Table S1** | Calculated intermolecular interaction energy components E_ele_, E_pol_, E_dis_, E_rep_, and E_tot_, in kJ mol^−1^ for the unique molecular pairs in the crystal structure, obtained using the CE-B3LYP/6-31G(d,p) energy model for CTD Form 1. | S10 |
| **Table S2** | Calculated intermolecular interaction energy components E_ele_, E_pol_, E_dis_, E_rep_, and E_tot_, in kJ mol^−1^ for the unique molecular pairs in the crystal structure, obtained using the CE-B3LYP/6-31G(d,p) energy model for CTD Form 2. | S12 |

**Figure S1.** Experimental and calculated PXRD patterns of CTD Forms I and II. Calculated patterns were derived from CCDC entries YUCCIJ (Form I) and YUCCIJ03 (Form II). Asterisks mark reflections of CTD Form I in the experimental pattern of Form II, indicating a bulk phase mixture with Form I as the minor phase.

**1.0 Rotational Barrier Analysis**

To investigate the conformational flexibility of the isolated molecules, a relaxed potential energy surface (PES) scan was performed for rotation about the C1–C9 bond using the M06-2X functional with both 6-311G(d,p) and aug-cc-pVDZ basis sets. The dihedral angle was varied from 0° to 360° in 10° increments. As shown in **Figure S2** and **S3**, both CTD-I and CTD-II exhibit a symmetric double-barrier potential with transition-state-like maxima near 120° and 300°, separated by stable minima around 0° (360°) and 190–200°. At the M06-2X/6-311+G(d,p) level, the calculated rotational barriers are 5.69 and 5.40 kcal mol⁻¹ for CTD-I, and 5.72 and 5.50 kcal mol^-1^ for CTD-II. The corresponding M06-2X/aug-cc-pVDZ calculations yield slightly lower barrier heights of 5.05 and 4.75 kcal mol^-1^ for CTD-I, and 5.08 and 4.80 kcal mol^-1^ for CTD-II. Despite this modest reduction (≈0.6–0.8 kcal mol^-1^), both basis sets reproduce the same stationary points, torsional pathway, and overall, PES topology, indicating that the conformational preferences are essentially independent of the basis set. These moderate rotational barriers suggest that rotation about the C1–C9 bond is restricted but remains thermally accessible at ambient temperature, thereby permitting conformational interconversion while preserving the preferred low-energy conformations. The 6-311G(d,p) basis set was employed throughout this study because it provides a reliable balance between computational cost and accuracy for M06-2X calculations on organic molecules, while the additional aug-cc-pVDZ calculations further confirm the robustness of the predicted rotational behavior.

**Figure S2.** Potential energy profiles for the rotation about the C8-C1-C9-C14 bond in CTD-I (A) and CTD-II (B) computed at the M06-2X/6-311G(d,p) level of theory. The dihedral angle was varied from 0° to 360° in 10° intervals and nearly overlapping energy profiles (C) indicating similar conformational preferences and rotational flexibility for both compounds.

**Figure S3**. Rotational energy profiles of CTD-I and CTD-II obtained by scanning the C8-C1-C9-C14 dihedral angle (10° intervals) at the M06-2X/6-311G(d,p) and M06-2X/aug-cc-pVDZ levels of theory. Relative energies are reported in kcal mol-1, showing comparable rotational barriers and conformational trends for both basis sets.

**Figure S4.** Gas-phase optimized geometries of CTD-I and CTD-II, including their stable conformer energy differences (in kcal/mol) and possible intramolecular interactions for each conformer.

**Figure S5.** Experimental Raman spectra of CTD Form I (black line) and Form II (red line); theoretical Raman spectra of CTD Form I (green line) and Form II (blue line).

**Figure S6.** Visualization of the Raman-active vibrational modes in the ring-breathing region for CTD Form I (A) and CTD Form II (B). The highlighted molecular fragments represent the principal atomic displacements associated with the breathing vibrations, while the yellow dashed lines indicate the intermolecular hydrogen-bonding interactions that influence the vibrational coupling and crystal packing.

**Figure S7.** Representative intermolecular interaction molecular pairs of CTD (a) Form I and (b) Form II.

**Figure S8.** Two-dimensional (2D) fingerprint plots illustrating the overall and contributions of intramolecular hydrogen-bonding contacts in both forms.

**Figure S9.** Two-dimensional (2D) fingerprint plots illustrating the overall and contributions of intermolecular hydrogen-bonding contacts in CTD Forms I.

**Figure S10.** Two-dimensional (2D) fingerprint plots illustrating the overall and contributions of intermolecular hydrogen-bonding contacts in CTD Forms II.

**Figure S11.** Representative intermolecular molecular pairs within a 10 Å cutoff distance around the reference molecule for CTD (a) Form I and (b) Form II.

The distances from a point on the Hirshfeld surface to the nearest atom inside and outside the surface are denoted as d_i_ and d_e_, respectively. The normalized contact distance, d_norm_, is then calculated using the following equation 1[1],

d_norm_ =+ (1)

In CrystalExplorer [1], the crystal lattice energy (E_lat_) is determined by summing the total interaction energies (E_tot_) of all symmetry-equivalent molecular pairs within the coordination cluster, weighted by the number of equivalent pairs (N), and divided by two to avoid double counting, as expressed by,

E_lat_ = 0.5 $\sum N*E_{tot}$	(2)

**Table S1.** Calculated intermolecular interaction energy components E_ele_, E_pol_, E_dis_, E_rep_, and E_tot_, in kJ mol^−1^ for the unique molecular pairs in the crystal structure, obtained using the CE-B3LYP/6-31G(d,p) energy model for CTD Form 1.

| **N** | **R** | **E_ele_** | **E_pol_** | **E_dis_** | **E_rep_** | **E_tot_** |
| --- | --- | --- | --- | --- | --- | --- |
| 1 | 13.54 | -0.5 | 0 | -0.2 | 0 | -0.7 |
| 1 | 11.36 | -3.9 | -0.1 | -0.5 | 0 | -4.7 |
| 2 | 15.54 | 1.5 | 0 | -0.2 | 0 | 1.4 |
| 1 | 13.11 | 0.6 | 0 | -0.4 | 0 | 0.2 |
| 2 | 12.82 | -0.5 | 0 | -0.2 | 0 | -0.7 |
| 2 | 12.45 | 2.1 | -0.1 | -0.6 | 0 | 1.7 |
| 2 | 8.86 | -1.9 | -0.4 | -2.1 | 0 | -4.2 |
| 1 | 14.67 | 0 | 0 | -0.1 | 0 | -0.1 |
| 1 | 15.79 | -1.2 | 0 | -0.2 | 0 | -1.5 |
| 2 | 6.23 | -16.1 | -9.2 | -49.9 | 41.8 | -41.4 |
| 2 | 16.93 | 0.4 | 0 | -0.1 | 0 | 0.2 |
| 1 | 12.22 | -3.7 | -0.7 | -2.6 | 0 | -6.7 |
| 1 | 19.37 | -0.3 | 0 | -0.1 | 0 | -0.4 |
| 1 | 12.93 | 0.8 | 0 | -0.3 | 0 | 0.6 |
| 1 | 8.03 | -68.1 | -15.9 | -17.2 | 50.9 | -67.2 |
| 2 | 16.36 | 0.5 | 0 | -0.2 | 0 | 0.4 |
| 2 | 14.52 | -0.2 | 0 | -0.1 | 0 | -0.3 |
| 1 | 8.55 | -3.1 | -0.5 | -15.8 | 20.5 | -4.7 |
| 2 | 8.39 | -12.2 | -4.4 | -21.6 | 13.2 | -26.8 |
| 1 | 10.96 | 2 | -0.1 | -0.7 | 0 | 1.4 |
| 2 | 11.82 | -5.7 | -2.3 | -10 | 8.1 | -11.5 |
| 2 | 14.36 | 0.1 | 0 | -0.2 | 0 | -0.1 |
| 1 | 10.95 | -48.1 | -8.9 | -22 | 37.1 | -53.7 |
| 2 | 19.27 | -0.1 | 0 | -0.1 | 0 | -0.2 |
| 2 | 16.71 | 0.1 | 0 | -0.1 | 0 | 0 |
| 1 | 17.53 | 0.5 | 0 | -0.1 | 0 | 0.4 |
| 2 | 19.53 | -0.8 | 0 | 0 | 0 | -0.9 |
| 1 | 13.9 | -4.3 | -1.3 | -11.2 | 8.2 | -10.1 |
| 1 | 21.2 | 0 | 0 | 0 | 0 | 0 |
| 2 | 16.77 | -1.1 | 0 | -0.1 | 0 | -1.3 |
| 1 | 13.28 | 1.1 | -0.1 | -0.3 | 0 | 0.8 |
| 1 | 8.81 | -76.6 | -18.8 | -45.2 | 86.3 | -80.9 |
| 1 | 7.21 | -4.7 | -1.5 | -25.5 | 17.6 | -17.4 |
| 1 | 10.13 | 0.7 | -0.1 | -1.1 | 0 | -0.2 |
| 1 | 8.25 | -7.7 | -7.4 | -23.9 | 18.3 | -23.3 |
| 1 | 15 | 4.3 | -0.1 | -0.2 | 0 | 4.2 |
| 1 | 17.73 | 0.3 | 0 | -0.3 | 0 | 0 |
| 1 | 16.23 | 0.9 | 0 | -0.1 | 0 | 0.8 |
| 1 | 12.97 | -0.3 | -0.2 | -1.4 | 0 | -1.7 |
| 1 | 10.4 | -0.9 | -0.3 | -1.5 | 0 | -2.4 |
| 1 | 12.76 | -0.7 | 0 | -0.3 | 0 | -1.1 |
| 1 | 9.69 | -5 | -0.4 | -1.1 | 0 | -6.6 |
| 1 | 14.84 | 1.2 | -0.1 | -0.1 | 0 | 1.2 |
| 1 | 15.56 | 0.1 | 0 | -0.1 | 0 | -0.1 |

**Table S2.** Calculated intermolecular interaction energy components E_ele_, E_pol_, E_dis_, E_rep_, and E_tot_, in kJ mol^−1^ for the unique molecular pairs in the crystal structure, obtained using the CE-B3LYP/6-31G(d,p) energy model for CTD Form 2.

| **N** | **R** | **E_ele_** | **E_pol_** | **E_dis_** | **E_rep_** | **E_tot_** |
| --- | --- | --- | --- | --- | --- | --- |
| 2 | 10.3 | -64.2 | -12.4 | -19.8 | 61.7 | -56.2 |
| 2 | 11.88 | -1.1 | -0.1 | -0.5 | 0 | -1.7 |
| 2 | 16.94 | -0.5 | 0 | -0.1 | 0 | -0.6 |
| 2 | 17.88 | -1 | 0 | -0.1 | 0 | -1.1 |
| 2 | 16.21 | 0.4 | 0 | -0.1 | 0 | 0.4 |
| 2 | 12.58 | -0.1 | 0 | -0.4 | 0 | -0.4 |
| 2 | 12.09 | 0.5 | 0 | -0.4 | 0 | 0.1 |
| 2 | 14.4 | -0.5 | 0 | -0.2 | 0 | -0.7 |
| 2 | 10.79 | -0.5 | -0.1 | -1.1 | 0 | -1.7 |
| 2 | 14.83 | -0.3 | 0 | -0.6 | 0 | -0.8 |
| 2 | 7.54 | -1.1 | -1.2 | -25.9 | 19.4 | -12.6 |
| 2 | 13.01 | 3.9 | -1.2 | -5.9 | 0 | -1.9 |
| 2 | 6.71 | -8.3 | -4.6 | -46.6 | 33.7 | -31.8 |
| 2 | 8.11 | -23.6 | -7.1 | -18.9 | 12.8 | -38.8 |
| 2 | 19.24 | -0.3 | 0 | -0.1 | 0 | -0.3 |
| 2 | 14.83 | -0.1 | 0 | -0.2 | 0 | -0.3 |
| 2 | 10.79 | -2 | -0.1 | -1 | 0 | -3.1 |
| 2 | 16.23 | -0.1 | 0 | -0.1 | 0 | -0.1 |
| 2 | 13.47 | 0.3 | 0 | -0.3 | 0 | 0 |
| 2 | 7.12 | 3.2 | -1 | -12.7 | 3.7 | -6.1 |
| 2 | 14.24 | 0.4 | 0 | -0.1 | 0 | 0.3 |
| 2 | 14.19 | 0.6 | 0 | -0.4 | 0 | 0.2 |
| 2 | 11.78 | -0.1 | 0 | -0.5 | 0 | -0.5 |
| 2 | 12.27 | 1.8 | -0.1 | -1.1 | 0 | 0.9 |
| 2 | 8.95 | -50.6 | -15 | -38 | 68.2 | -55.5 |
| 2 | 14.19 | -0.2 | 0 | -0.2 | 0 | -0.4 |
| 2 | 17.96 | 0 | 0 | -0.1 | 0 | -0.2 |
| 2 | 18.29 | 0.2 | 0 | -0.1 | 0 | 0.2 |
| 2 | 15.5 | 0.1 | 0 | -0.1 | 0 | 0 |
| 2 | 15.7 | 0.8 | -0.1 | -0.2 | 0 | 0.6 |

**Reference**

1. Spackman, P. R., et al. (2021) CrystalExplorer: a program for Hirshfeld surface analysis, visualization and quantitative analysis of molecular crystals J. Appl. Cryst. 54, 1006–101. https://doi.org/10.1107/S1600576721002910
